# Synergistic combination of valproic acid and oncolytic parvovirus H-1PV as a potential therapy against cervical and pancreatic carcinomas

**DOI:** 10.1002/emmm.201302796

**Published:** 2013-09-17

**Authors:** Junwei Li, Serena Bonifati, Georgi Hristov, Tiina Marttila, Séverine Valmary-Degano, Sven Stanzel, Martina Schnölzer, Christiane Mougin, Marc Aprahamian, Svitlana P Grekova, Zahari Raykov, Jean Rommelaere, Antonio Marchini

**Affiliations:** 1 Infection and Cancer Program, Tumor Virology Division (F010), German Cancer Research Center (DKFZ)Heidelberg, Germany; 2 Institut d' Anatomie et Cytologie Pathologiques, Hopital Jean MinjozBesançon, France; 3 Department of Biostatistics, German Cancer Research Center (DKFZ)Heidelberg, Germany; 4 Protein Analysis Facility, German Cancer Research Center (DKFZ)Heidelberg, Germany; 5 CHU Saint-Jacques, Laboratoire de Biologie Moléculaire et CellulaireBesançon, France; 6 Institut de Recherche Contre les Cancers de l'Appareil DigestifStrasbourg, France

**Keywords:** H-1PV, pancreatic ductal adenocarcinomas, parvovirus NS1 protein, valproic acid, viral oncotherapy

## Abstract

The rat parvovirus H-1PV has oncolytic and tumour-suppressive properties potentially exploitable in cancer therapy. This possibility is being explored and results are encouraging, but it is necessary to improve the oncotoxicity of the virus. Here we show that this can be achieved by co-treating cancer cells with H-1PV and histone deacetylase inhibitors (HDACIs) such as valproic acid (VPA). We demonstrate that these agents act synergistically to kill a range of human cervical carcinoma and pancreatic carcinoma cell lines by inducing oxidative stress, DNA damage and apoptosis. Strikingly, in rat and mouse xenograft models, H-1PV/VPA co-treatment strongly inhibits tumour growth promoting complete tumour remission in all co-treated animals. At the molecular level, we found acetylation of the parvovirus nonstructural protein NS1 at residues K85 and K257 to modulate NS1-mediated transcription and cytotoxicity, both of which are enhanced by VPA treatment. These results warrant clinical evaluation of H-1PV/VPA co-treatment against cervical and pancreatic ductal carcinomas.

## INTRODUCTION

Despite great progress in cancer therapy over the years, conventional treatment of pancreatic, liver, lung, brain and advanced cervical cancers remains largely ineffective (American Cancer Society, 2012). Hence novel therapeutic strategies are needed. Oncolytic viruses, which exploit cancer-specific vulnerabilities to kill cancer cells while sparing normal cells, are fast emerging as promising tools for fighting cancer (Breitbach et al, 2011; Russell et al, 2012). No less than 12 different oncolytic viruses are currently undergoing phase I-III clinical trials against various malignancies (Russell et al, 2012). One of them is the rat parvovirus H-1PV, attractive because of its anticancer activities demonstrated in various *in vitro* cell systems and animal models (Rommelaere et al, 2010). H-1PV is currently tested for safety and first indication of anticancer efficacy in a phase I/IIa clinical trial involving patients with glioblastoma multiforme (Geletneky et al, 2012).

The parvovirus genome consists of a single stranded DNA molecule of approximately 5100 bases including two promoters, P4 and P38 which regulate expression of the viral nonstructural proteins (NS1 and NS2) and capsid proteins (VP1 and VP2), respectively (Nuesch et al, 2012). H-1PV infection induces oxidative stress causing DNA damage, cell cycle arrest and apoptosis. These events are mediated by the NS1 protein that alone is sufficient to trigger the whole cell death cascade induced by the complete virus (Hristov et al, 2010). Besides being the major effector of parvovirus cytotoxicity, the NS1 protein plays other key roles in the virus life-cycle as a regulator of viral DNA replication and gene expression (Nuesch, 2006). It binds as an oligomer to DNA, notably to the (ACCA)_2–3_ motifs present within the P4 and P38 promoters (Cotmore et al, 1995). However, the mechanisms regulating NS1 functions and controlling the H-1PV life cycle remain largely uncharacterized (Nuesch et al, 2012).

Due to their genetic heterogeneity, it is likely that some of the cancer cells within a tumour will have a different sensitivity to H-1PV. It is therefore important to reinforce the antineoplastic activity of the virus in order to improve its clinical outcome in such a scenario.

This can be achieved by developing combination strategies based on virus and other anticancer agents that increase cancer cell killing while minimizing toxic side effects.

Histone deacetylase inhibitors (HDACIs) hold much promise in cancer therapy, because they reactivate transcription of multiple genes and cause cancer cell growth inhibition, differentiation and death (Minucci & Pelicci, 2006). Two HDACIs, suberoylanilide hydroxamic acid (SAHA, Vorinostat, Zolinza®) and romidepsin are used to treat cutaneous T-cell lymphoma (Rodriguez-Paredes & Esteller, 2011). Furthermore, over 80 clinical trials are in progress to test the efficacy of 12 different HDAC inhibitors, used as monotherapy or in combination with conventional chemotherapy against a wide variety of tumours (Lee et al, 2012). In particular, valproic acid (VPA), already used clinically as an antiepileptic agent, is being tested as anticancer agent in a phase III clinical trial for cervical carcinomas (Coronel et al, 2010; Gottlicher et al, 2001). HDACIs have also been shown to reinforce the cytotoxicity of oncolytic viruses, including the vesicular stomatitis virus (VSV; Alvarez-Breckenridge et al, 2012), herpes simplex virus (HSV; Otsuki et al, 2008) and adenoviruses (VanOosten et al, 2007), by repressing the expression of host cell genes involved in the antiviral immune response or by stimulating the expression of genes required for the viral life cycle (Nguyen et al, 2010).

In the present study we have investigated whether HDACIs might enhance the antitumour activities of H-1PV against cervical carcinoma (CC) and pancreatic ductal adenocarcinoma (PDAC). We show that H-1PV and VPA act synergistically to kill tumour cells both *in vitro* and *in vivo*. We also show for the first time that NS1 is acetylated at residues K85 and K257 and that addition of VPA correlates with an enhanced rate of NS1 acetylation. We provide important evidence that acetylation modulates NS1 functions. Indeed, amino-acid substitution of the two acetylation sites strongly impairs NS1-mediated viral gene transcription, viral replication and cytotoxicity. On the contrary, VPA-induced hyper-acetylation of NS1 converts the protein into a more active polypeptide. We call for clinical evaluation of H-1PV/VPA co-treatment against CC and PDAC, as the synergy of H-1PV and VPA should allow the use of these agents at lower concentrations, with increased safety and therapeutic effect.

## RESULTS

### VPA synergizes with H-1PV to kill cancer cells

**Figure 1:**
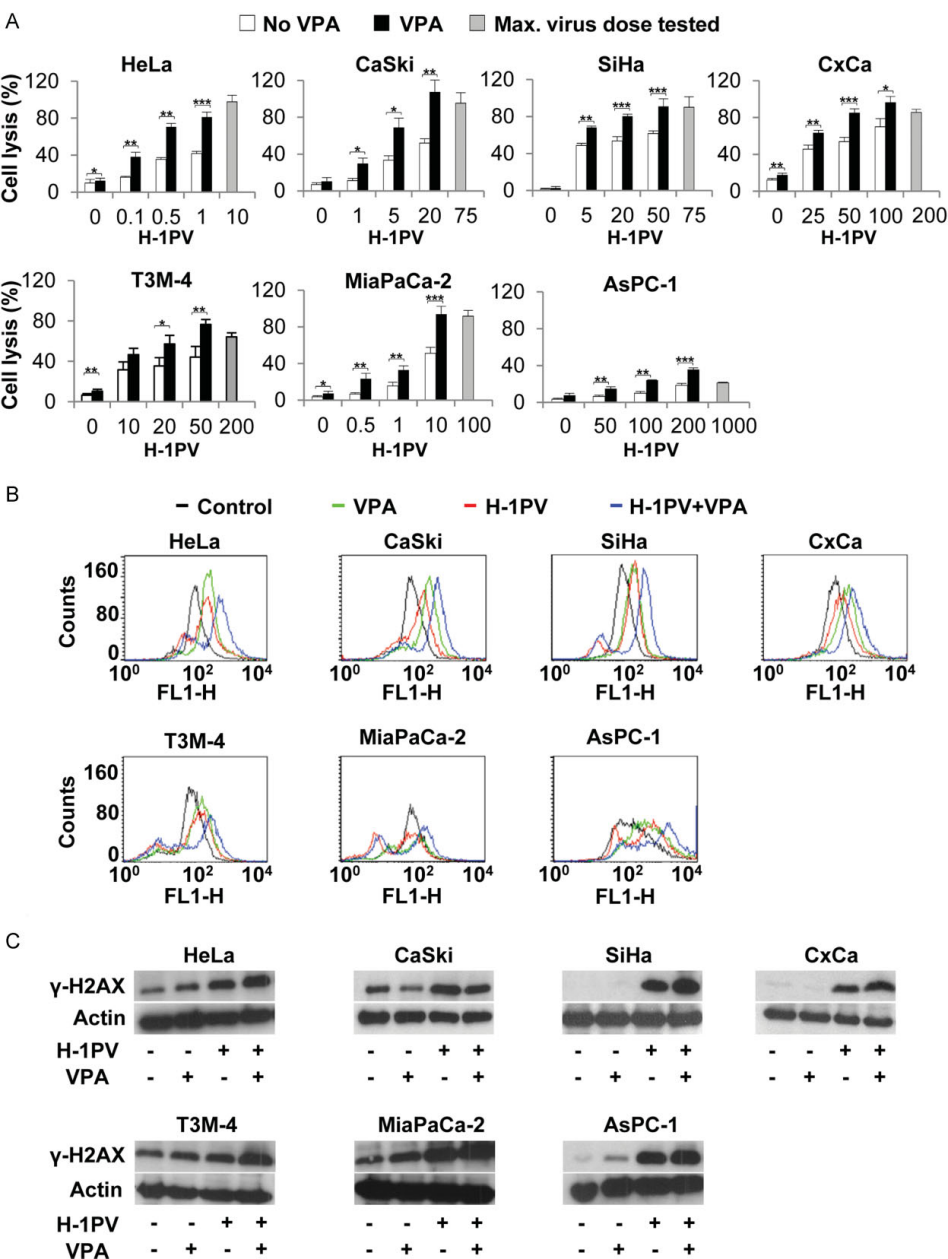
H-1PV and VPA synergize to induce cell death, oxidative stress and DNA damage in cervical and pancreatic derived cancer cell lines Source data is available for this figure in the Supporting Information. Analysis of cell lysis. Cancer cell lines were seeded into 96-well plates and infected with H-1PV at the indicated MOI (pfu/cell) in combination or not with VPA (1 mM). The maximum viral dose tested is shown in grey. Cell lysis was measured after 72 h by means of the LDH assay, as described under Materials and Methods Section. Columns show average cell lysis values with standard deviation bars. Results from a typical experiment performed in quadruplicate are shown. *p<0.05; **p<0.01; ***p<0.001 as calculated by two sample Welch *t*-test and adjustment for multiple tests with the Bonferroni method.ROS content. Representative FACS-plots of DCFH-DA-stained cancer cells, untreated (black) or treated with VPA alone (green), with H-1PV alone (red) or with both H-1PV and VPA (blue). FACS analysis of cervical and pancreatic cancer cells was performed, at 24 and 48 h post-treatment respectively, as described in Materials and Methods Section. Every experiment was performed at least in triplicate and repeated at least twice. A minimum of 20,000 events was acquired.DNA damage. Western blot analysis of the levels of the DNA damage marker protein phosphorylated H2AX (γ-H2AX) in cervical and pancreatic cancer cells left untreated or treated with VPA, H-1PV, or both. 20 µg total cell lysate were used for analysis. Actin was used as a loading control.

We first investigated the oncotoxicity of H-1PV/VPA co-treatment using cell lines derived from CC [HeLa, CaSki, SiHa and early-passage tumour cell cultures (CxCa)] and PDAC (T3M-4, MiaPaCa-2 and AsPC-1). Among the selected cell lines only HeLa cells sustain efficient viral multiplication, while the other cell lines are low permissive for virus replication (Dempe et al, 2010; our unpublished results). Two doses of VPA were selected based on the concentrations that have been established in patients with epilepsy: 0.5 mM (close to the typical therapeutic serum concentration of 0.6 mM) and 1 mM (close to the upper limit of antiepilectic range of 0.9 mM; Li et al, 2005b). Seventy-two hours after infection with H-1PV, the lactate dehydrogenase (LDH) assay revealed virus-dose-dependent killing in all of the lines tested but the sensitivity was dependent on the cell line. While HeLa cells showed massive death at a relatively low multiplicity of infection [MOI of 10 plaque forming units (pfu) per cell], all the other cell lines showed low sensitivity to viral infection (approximately 70% killing of CaSki, SiHa, CxCa, T3M-4 and MiaPaCa-2: at MOIs of 75–200 pfu/cell), or appeared quite resistant (AsPC-1: only 20% mortality at a MOI of 1000 pfu/cell; Fig 1A). No apparent or modest cytotoxicity was observed with VPA alone at the concentrations used. Remarkably, H-1PV-mediated cell lysis was significantly enhanced in the presence of VPA in all cell lines analysed, increasing in some cases by 50% to more than 100% (two-way ANOVA with interaction; all *p*<0.001; Fig 1A and Supporting Information Fig S1 and Table S1). Under similar conditions co-treatment did not affect normal primary cells (Supporting Information Fig S2).

Reactive oxygen species (ROS) are important mediators of H-1PV cytotoxicity and causes of DNA damage (Hristov et al, 2010). VPA has also been shown to induce production of pro-apoptotic ROS (Minucci & Pelicci, 2006). We therefore investigated whether the enhanced H-1PV cytotoxicity observed in the presence of VPA might correlate with increased ROS accumulation and DNA damage. In agreement with previous results obtained in HeLa cells (Hristov et al, 2010), H-1PV was found to induce ROS accumulation in all cell lines tested except the pancreatic carcinoma derived line MiaPaCa-2. Strikingly, while cells subjected to VPA treatment or H-1PV infection alone showed similar levels of ROS accumulation, all cell lines tested showed enhanced ROS accumulation when treated with both agents (Fig 1B). This accumulation was generally associated with increased levels of phosphorylated histone H2AX (γ-H2AX), a hallmark of DNA damage response, the latter levels being higher after combined treatment than after treatment with H-1PV or VPA alone (Fig 1C). Then we tested the effect of combining H-1PV with another HDACI, namely sodium butyrate (NaB). Like VPA, sublethal doses of NaB were found to boost parvovirus-mediated cytotoxicity in HeLa cells. This enhanced cytotoxicity was associated with increased oxidative stress and DNA damage (Supporting Information Fig S3).

### VPA enhances the cytotoxic activities of the H-1PV NS1 protein

**Figure 2:**
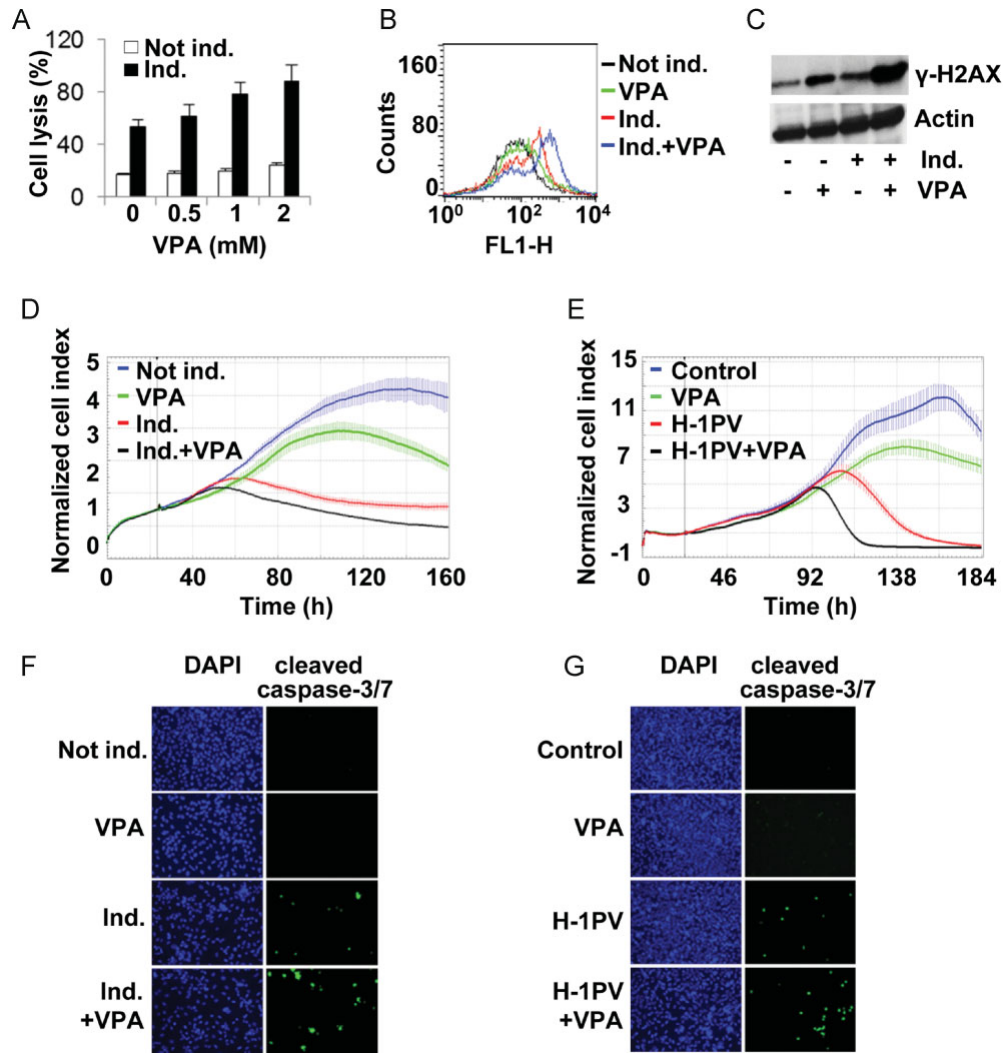
VPA increases NS1-mediated cytotoxicity Source data is available for this figure in the Supporting Information. LDH release. HeLa-NS1 stable cell line was grown with (Ind.) or without (Not ind.) doxycycline (1 µg/ml) and with VPA at the indicated concentration (mM) for 96 h before to be processed for LDH assay.ROS content. HeLa-NS1 cells were grown in medium supplemented (Ind.) or not (Not ind.) with doxycycline and treated with VPA (1 mM) for 24 h before ROS content analysis.DNA damage. Lysates from cells treated as describe in panel B were subjected to Western blot analysis using antibodies specific for γ-H2AX and actin (loading control).Cell proliferation of NS1 expressing cells. HeLa-NS1 cells were induced (Ind.) or not (Not ind.) with doxycycline and grown in the presence or absence of VPA (0.5 mM). Cell proliferation was monitored in real time with the xCELLigence system as described in Materials and Methods Section. The vertical grey bar indicates the time of treatment.Cell proliferation of H-1PV infected HeLa cells. HeLa cells were mock treated (control), or treated with VPA (1 mM) or H-1PV (MOI 0.01 pfu/cell) or both and analysed as described in panel D.Analysis of apoptosis of NS1 expressing cells. HeLa-NS1 cells were treated as in panel B for 24 h before being loaded with 5 µM CellEvent™ Caspase-3/7 Green Detection Reagent for detection of active caspases 3 and 7. Nuclei were visualized by DAPI staining.Analysis of apoptosis of H-1PV infected HeLa cells. HeLa cells were either mock-treated (control) or infected with H-1PV (MOI 1 pfu/cell) in the presence or absence of VPA (1 mM) and treated as described in panel F.

In HeLa cells, NS1 alone is sufficient to induce intracellular ROS accumulation and DNA damage leading to apoptosis and cell lysis (Hristov et al, 2010). We therefore assumed that the enhanced H-1PV cytotoxicity observed in the presence of VPA or NaB might be due to stimulation of NS1 activities. To answer this question, we used a stable cell line (HeLa-NS1) characterized by doxycycline (dox) inducible expression of the NS1 gene (in the absence of any other viral component) (Hristov et al, 2010). These cells were exposed or not to VPA or NaB and the LDH assay was used to evaluate cell lysis. Both HDACIs were found to enhance NS1-triggered cell lysis (Fig 2A and Supporting Information Fig S4A). As in cells infected with H-1PV, this was associated with increased ROS accumulation and DNA damage (Fig 2B and C and Supporting Information Fig S4B and C). We next examined in real time the effect of VPA on proliferation of NS1-expressing cells in comparison to the proliferation of H-1PV infected cells. Again we found that the intrinsic cytotoxicity of both NS1 and of the full H-1PV virus was enhanced by the HDAC inhibitor (Fig 2D–E, compare the red and black curves). Analysis of apoptosis confirmed that VPA enhances the cytotoxic activity of both NS1 and H-1PV (Fig 2F and G). A similar increase in the induction of apoptosis was also observed in H-1PV/NaB co-treated cells (data not shown). Altogether these results indicate that above HDACIs increase NS1-mediated cytotoxicity.

### NS1 is acetylated and VPA-enhanced NS1 acetylation correlates with increased NS1 transcriptional activity and virus production

**Figure 3:**
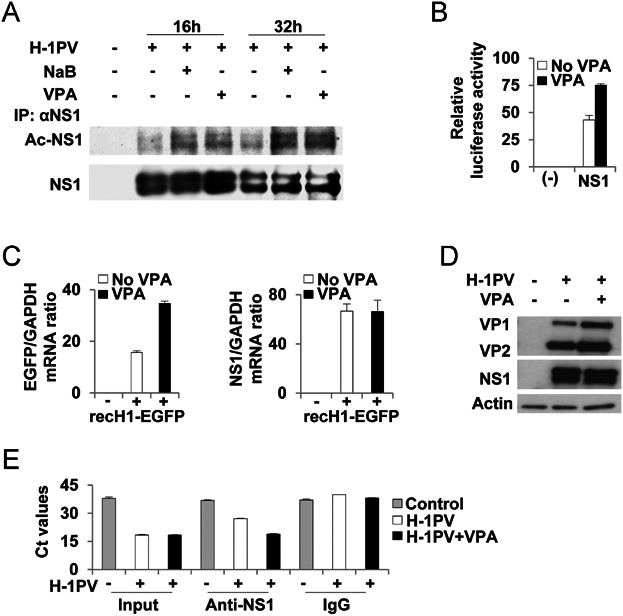
VPA treatment of HeLa cells increases NS1 acetylation and NS1-dependent transcriptional activity Source data is available for this figure in the Supporting Information. The H-1PV nonstructural protein NS1 is acetylated *in vivo*. Mock or H-1PV-infected HeLa cells (MOI of 0.5 pfu/cell) were treated with VPA (1 mM) or NaB (1 mM) for 16 or 32 h before to be lysed. Immunoprecipitated NS1 was analysed by Western blotting with antibody against acetylated lysines. For detection of total NS1 protein, the membrane was stripped and re-probed with anti-NS1 antiserum.VPA treatment enhances NS1 transcriptional activities. HeLa cells were transfected with pCDNA4/TO-Flag-HA-NS1-H1 (NS1) or pCDNA4/TO empty vector (−), together with the pGL3-H-1PV-P38 and the pRL-TK plasmid. Transfected cells were grown in a medium containing or not containing VPA (1 mM) for 48 h. Cells were then lysed and subjected to Dual Luciferase Assay. Values represent the mean ratios of *firefly* to *Renilla* luciferase activity with standard deviation bars, for three replicates.VPA treatment increases NS1-driven gene expression. HeLa cells seeded into 6-cm dishes in a medium supplemented or not with VPA (1 mM) were mock-treated (−) or infected with an H-1 recombinant parvovirus (recH1-EGFP) at a MOI 1 transduction unit per cell (+). The recH-1-EGFP vector carries the *EGFP*-encoding transgene under the control of the NS1-inducible viral promoter P38. After 48 h, cells were collected, total RNA was isolated and reverse transcribed. Quantitative PCR was carried out using specific primers for *EGFP*, *NS1* and *GAPDH* (used as a housekeeping gene). The EGFP and NS1 mRNA expression levels were normalized to those of GAPDH mRNA. Normalized expression values presented are means with standard deviation bars from three measurements.VPA treatment increases viral protein synthesis. HeLa cells were infected with H-1PV (MOI of 1 pfu/cell) and grown for 48 h in the presence or absence of VPA (1 mM). Cell lysates were analysed by Western blotting for the presence of viral NS1 and capsid (VP1 and VP2) proteins. Actin was used as a loading control.VPA enhances binding of NS1 to the P38 promoter. Chromatin immunoprecipitation and PCR amplification of the precipitated viral P38 promoter region was performed as described in Materials and Methods Section. Results are presented as average Ct values with standard deviation bars, computed from duplicate measurements per experimental conditions.

**Figure 4:**
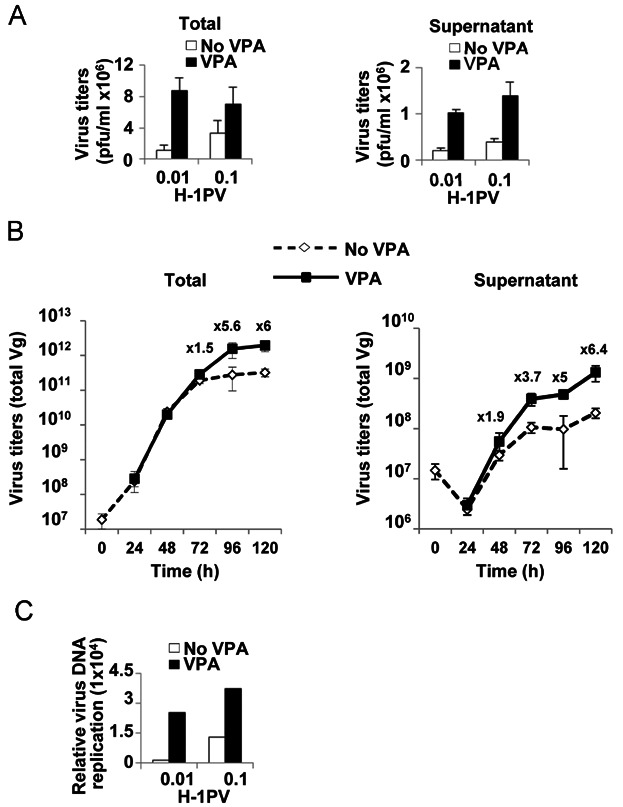
VPA treatment increases production of progeny virions Plaque assay. 1 × 10^6^ HeLa cells were infected with H-1PV (MOI of 0.01 or 0.1 pfu/cell) in the presence (black) or absence (white) of VPA (1 mM) for one production cycle. At 96 h post-infection, the plaque assay was used to determine the number of viral particles in total cell lysates and culture medium. Average values from a typical experiment performed in triplicate are shown with standard deviation bars.Quantitative real time-PCR. HeLa cells were treated as in panel A. At the indicated time points, cells were harvested and viral particles recovered from total cell lysates (left panel) or culture medium (right panel). Viral DNA was quantified by real time PCR. Numbers on top of the curves indicate the fold increase in virus titers in the presence *versus* absence of VPA.Southern blotting. Viral DNA was extracted from crude lysates of cells treated as described in panel A and analysed by Southern blotting. DNA samples were fractionated by agarose gel electrophoresis, transferred to a Hybond N nylon membrane and hybridized with an NS1-specific DNA probe. Viral replicative-form DNA was quantified by means of a PhosphorImager.

The above results indicate that VPA enhances NS1 cytotoxicity, suggesting that acetylation may modulate NS1 activities. We therefore checked whether NS1 might undergo acetylation. H-1PV-infected HeLa cells were grown for 16 or 32 h in the presence or absence of VPA or NaB. Then cell extracts were prepared and the NS1 protein immunoprecipitated. Western blot analysis revealed a small but consistent fraction of the immunoprecipitated NS1 to be acetylated. This fraction was found to be increased in response to VPA or NaB treatment in a time-dependent fashion (Fig 3A). Besides having cytotoxic activities, NS1 is also the major regulator of viral DNA replication and gene expression. It can activate the H-1PV P38 late promoter which governs expression of the VP gene encoding the capsid proteins (Nuesch et al, 2012). Remarkably, both VPA and NaB were found to enhance NS1 transcriptional activity (Fig 3B and Supporting Information Fig S5A). To confirm these results, we infected HeLa cells with a recombinant H-1PV in which the VP gene was replaced with the EGFP encoding gene (recPV-EGFP; El-Andaloussi et al, 2011). VPA was found to enhance NS1-triggered EGFP but not NS1 expression (Fig 3C). Consistently, cells infected with wild-type H-1PV showed increased levels of VP capsid proteins in the presence of VPA or NaB, while levels of NS1 remained unchanged (Fig 3D and Supporting Information Fig S5B). This constitutes additional evidence that NS1 transcriptional activity is stimulated by HDACIs. In a chromatin immunoprecipitation (ChIP) assay, we found VPA to enhance the ability of NS1 to bind to the P38 promoter (Fig 3E). We hypothesized that increased viral gene expression could result in higher yields of progeny virus in VPA co-treated cells. In agreement with our hypothesis, co-treatment with VPA was found to increase the yield of virus production (up to eight fold at a MOI of 0.01 pfu/cell after 120 h) and to enhance the release of progeny viruses from infected cells (Fig 4A and B). Time course experiments confirmed increased virus multiplication in the presence of VPA. Interestingly, while viral titers in the absence of VPA reached a plateau after 72 h, VPA stimulated virus production beyond that limit and promoted more efficient viral release (Fig 4B). In agreement with these results, we also observed increased viral DNA replication in H-1PV/VPA co-treated HeLa cells (Fig 4C). Virus production stimulation by VPA was also found in SiHa and CaSki cells (Supporting Information Fig S6). We conclude that VPA stimulates NS1 transcriptional activities and increases virus production and release. This effect is likely to contribute to the increased cytotoxicity observed in H-1PV/VPA co-treated cells.

### Acetylation modulates NS1 cellular activities

**Figure 5:**
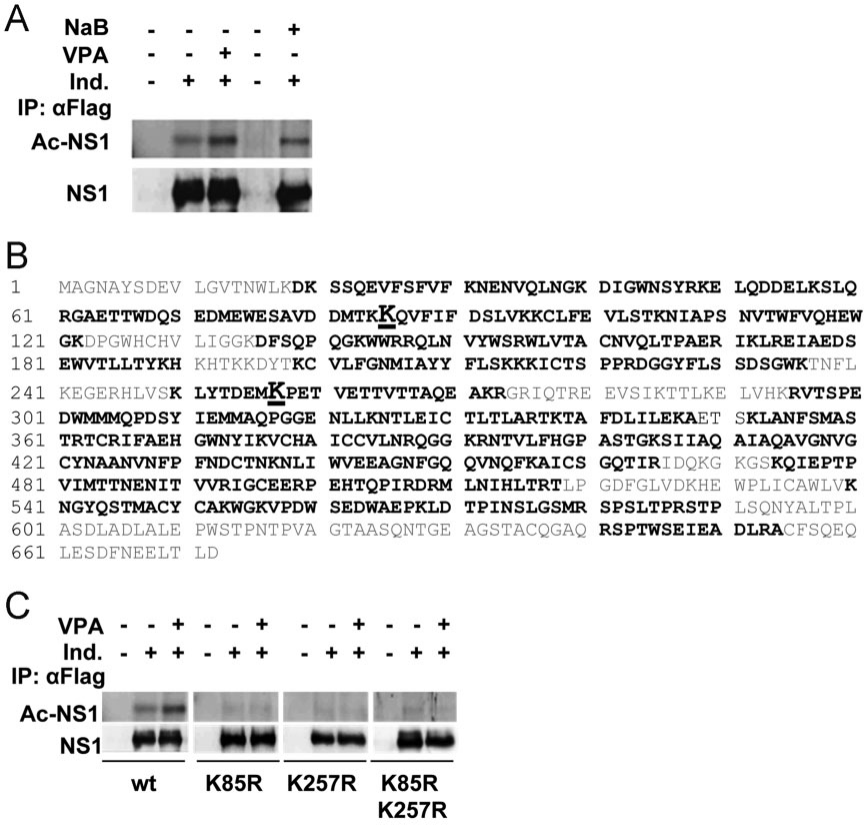
NS1 is acetylated at K85 and K257 Source data is available for this figure in the Supporting Information. Treatment with VPA or NaB increases NS1 acetylation. HeLa-NS1 cells were grown in 15-cm dishes in a medium containing (Ind. +) or not (Ind. −) doxycycline with or without VPA or NaB (both used at the concentration of 1 mM) for 24 h. Immunoprecipitation was performed on total cell lysates using an anti-Flag antibody recognizing the Flag-tagged NS1 protein. Immunoprecipitated proteins were subjected to SDS–PAGE and analysed for the presence of acetylated NS1 with an antibody recognizing acetylated lysines. Membrane was then stripped and further analysed for total NS1 protein levels using an anti-NS1 polyclonal antiserum.Identification of two NS1 acetylation sites. The stable cell line HeLa-NS1 was induced with doxycycline (1 µg/ml) and grown in the presence of VPA (1 mM). NS1 was immunoprecipitated from total cell lysates with monoclonal anti-FLAG antibody, in-gel purified, trypsin-digested and then subjected to MS analysis. The complete amino-acid sequence of the H-1PV NS1 protein is shown with, in grey, the amino-acids not covered by the MS analysis and, in black, those analysed by MS. The two identified acetylation sites K85 and K257 are highlighted in black boldface underlined characters.The K85R and K257R mutations reduce the NS1 acetylation level. The two K residues identified as NS1 acetylation sites were converted to R (to mimic their non-acetylated state), and pCDNA4/TO plasmids encoding singly or doubly mutated NS1 (FLAG/HA-tagged at its N-terminus) were used to generate stable cell lines. 5 × 10^6^ HeLa-NS1wild-type (wt), HeLa-NS1-K85R, HeLa-NS1-K257R and HeLa-NS1-K85R-K257R cells were either not induced (−) or induced (+) with doxycycline (for NS1 expression) and grown in the presence or absence of VPA (1 mM) for 24 h before harvest. The NS1 proteins were immunoprecipitated from total cell lysates with anti-FLAG antibody and assayed for acetylation as described in Fig 3A.

**Figure 6:**
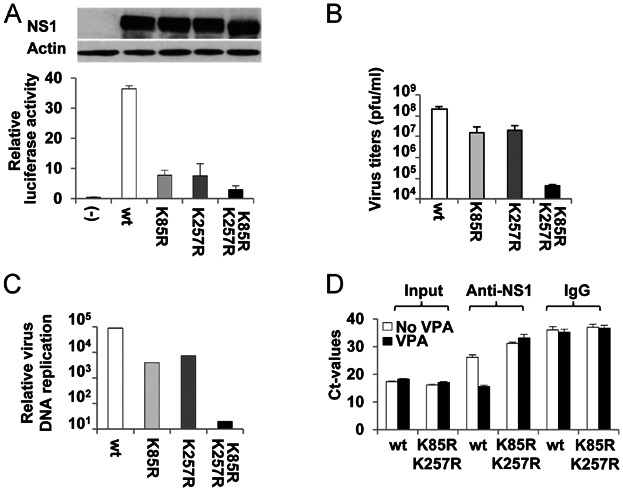
Acetylation modulates NS1 transcriptional activities The K85R and K257R mutations decrease NS1-dependent transactivation of the P38 promoter. HeLa cells were co-transfected with pGL3-H-1PV-P38, pRL-TK luciferase and the pCDNA4/TO plasmid, either empty (−) or expressing a wild-type NS1 (wt) or an acetylation-defective mutant form of this protein. 48 h post-transfection, the cells were processed for the firefly Dual-Luciferase Assay. Western blot analysis was performed with total cell lysates from these cultures in order to ascertain equal NS1 protein levels.Source data is available for this figure in the Supporting Information.Mutation of both NS1 acetylation sites strongly impairs H-1PV production. NB324K cells were infected with the same amount (100 viral genome/cell) of either H-1PV wild-type (wt) or acetylation-defective H-1PV-K85R, H-1PV-K257R, or H-1PV-K85R-K257R for a 6-day round of virus amplification. Production of progeny viral particles was determined by plaque assay. Average values from a representative experiment performed in triplicate are shown with standard deviation bars.Mutation of both NS1 acetylation sites strongly reduces H-1PV DNA replication. Viral DNA from HeLa cells infected with the indicated virus was analysed by Southern blot analysis and quantified with a PhosphorImager.Mutation of the NS1 acetylation sites strongly impairs the capacity of the protein to bind to the H-1PV P38 promoter. HeLa cells were infected with either H-1PV wild-type (wt) or with the H-1PV-NS1-K85R-K257R mutant and grown with (black bars) or without (white bars) VPA (1 mM) for 24 h before processing for ChIP assays as described in Fig 3E.

**Figure 7:**
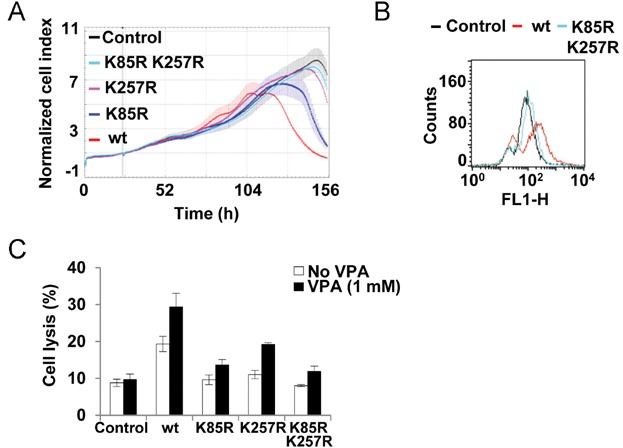
Acetylation modulates NS1 cytotoxic activities Mutation of the NS1 acetylation sites decreases H-1PV cytotoxicity. Cells were seeded into a 96-well E-plate and infected with mutant or wild-type (wt) viruses at the same MOI (10 Vg/cell). Cell proliferation was monitored in real time with the xCELLigence system as described in Fig 2D. The vertical grey line indicates the time of treatment.Mutation of both NS1 acetylation sites decreases H-1PV-triggered intracellular ROS accumulation. HeLa cells were infected with 500 Vg/cell of H-1PV wild-type (wt) or H-1PV-NS1-K85R-K257R mutant virus. 24 h after infection, cells were loaded with DCFH-DA, harvested and analysed by FACS for ROS content as described in Fig 1B.Mutation of the NS1 acetylation sites decreases H-1PV-triggered cell lysis. HeLa cells were infected with H-1PV wild-type (wt) or mutant derivatives (H-1PV-K85R, H-1PV-K257R, H-1PV-K85R-K257R) and grown for 72 h in the presence or absence of VPA before being processed for the LDH assay as described under Materials and Methods Section. Results shown are average cell lysis values with standard deviation bars, calculated from four replicates per experimental condition.

The above results prompted us to use mass spectrometry (MS) to identify the acetylation sites in NS1. Induced HeLa-NS1 cells were grown in the presence of VPA or NaB and NS1 was immunoprecipitated from total protein extracts. As observed in virus-infected cells, the fraction of acetylated NS1 increased in induced HeLa-NS1 cells grown in the presence of HDACIs (Fig 5A). After 2D-gel electrophoresis, purified NS1 was trypsin-digested and processed for MS. We analysed 36 out of 48 lysines present in the NS1 amino-acids sequence and identified two acetylation sites corresponding to lysine (K) 85 and K257 (Fig 5B). To further test the relevance of acetylation for NS1 functions, we examined the effect of substituting R for K at one or both sites (so to prevent acetylation) in new stable cell lines expressing the corresponding mutant construct. Each substitution was found to strongly reduce the fraction of acetylated NS1, confirming that these K residues are NS1 acetylation sites (Fig 5C). We then compared the transcriptional activity of the acetylation-defective NS1 mutants with that of the wild-type protein. In cells expressing the double mutant, we observed a more than 20-fold decrease in NS1-mediated transactivation of the P38 promoter (Fig 6A). Next, the same mutations were introduced into the viral genome. Mutant viruses were produced at lower titers than the wild-type virus especially in the case of the double mutant virus (a more than three logs reduction was observed; Fig 6B). Accordingly, a dramatic decrease in viral DNA replication was observed in the case of the double mutant virus (Fig 6C). In ChIP assays, we found the NS1 double mutant to have a reduced ability to bind to the P38 promoter (Fig 6D). Finally, we compared the cytotoxic effects of the NS1 mutant and wild-type viruses. Both single-mutant H-1PV viruses showed reduced cytotoxicity towards infected cells in comparison with wild-type virus, while the double mutant showed no apparent cytotoxicity (Fig 7A). Decreased virus cytotoxicity correlated with reduced ROS accumulation (Fig 7B and data not shown) and reduced cell lysis, which could be only partially restored by VPA addition (Fig 7C). Altogether, these results support the view that acetylation plays a key role in modulating NS1 activities and that VPA exerts its synergistic action by increasing the NS1 acetylation status.

### H-1PV/VPA co-treatment leads to complete eradication of established tumours

**Figure 8:**
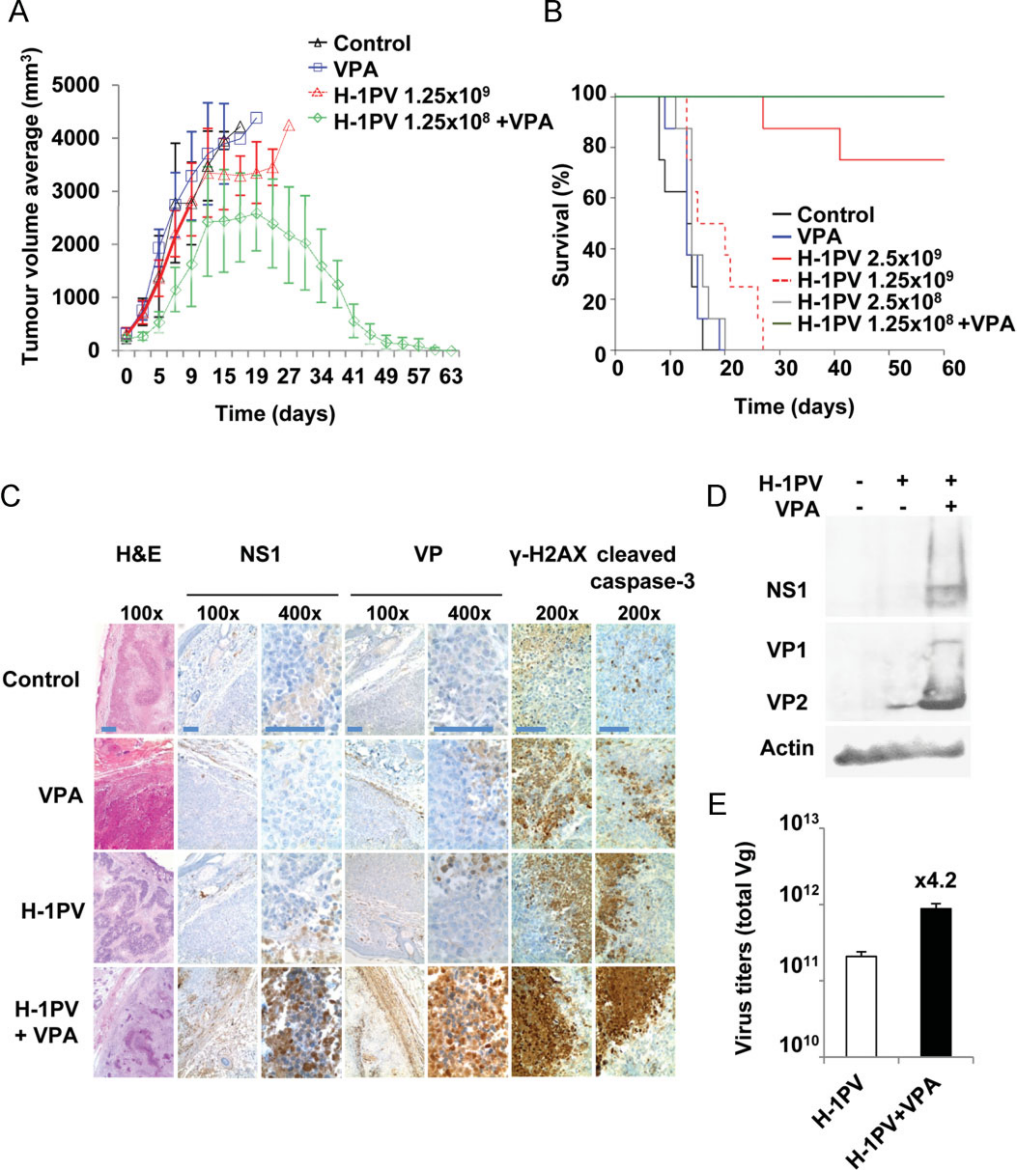
H-1PV/VPA co-treatment leads to complete remission of established HeLa tumours Source data is available for this figure in the Supporting Information. Analysis of tumour growth. Animals (*n* = 8 per group) were treated with either PBS (control), VPA or a combination of both agents as described in Materials and Methods Section. Other animal groups are presented in Supporting Information Fig S7. The data shown represent average tumour volumes with standard deviation bars.Graphical summary of Kaplan–Meier survival analysis of tumour-bearing nude rats treated as indicated. Animals were sacrificed when the tumour reached the maximum tolerable size of 4000 mm^3^ according to law. The difference between H-1PV/VPA co-treatment and treatment with H-1PV alone was statistically significant (*p* = 0.0002 as calculated with the two-sided log-rank test and adjusted for multiple testing with the Bonferroni method). Other results from the statistical analysis are shown in Supporting Information Table S2.H-1PV/VPA co-treatment induces DNA damage and apoptosis *in vivo*. Two rats of each treatment group were sacrificed on day 19. Tumours were fixed, sectioned and examined morphologically either by H&E staining or by immunochemistry using antibodies against NS1, VP capsid proteins, γ-H2AX (a DNA damage marker), or active cleaved caspase-3 (an apoptosis marker). Scale bar = 50 µm.Higher levels of NS1, VP1 and VP2 viral proteins in tumour samples from VPA co-treated animals. Tumour-bearing animals (*n* = 3) treated with H-1PV alone (total virus dose of 3.6 × 10^9^ Vg/animal corresponding to 1.5 × 10^8^ pfu/animals subdivided into two subdoses given at days 0 and 7) or co-treated with H-1PV and VPA (100 mg/kg) were sacrificed at day 14. Complete tumours were resected, homogenised and lysed. 20 µg of total cellular extracts were analysed by SDS–PAGE for the presence of the viral proteins. Actin was used as a loading control.VPA-enhanced virus multiplication *in vivo*. Viral particles were recovered from aliquots of tumour extracts from animals treated as in panel D, purified by using the QIAamp MinElute Virus Spin Kit (Qiagen) and quantified by real time qPCR. Columns represent the average amounts of the virus recovered from the tumours of three animals with standard deviation bars. Numbers on top of the columns indicate the fold increase in virus titers in the presence *versus* absence of VPA.

**Figure 9:**
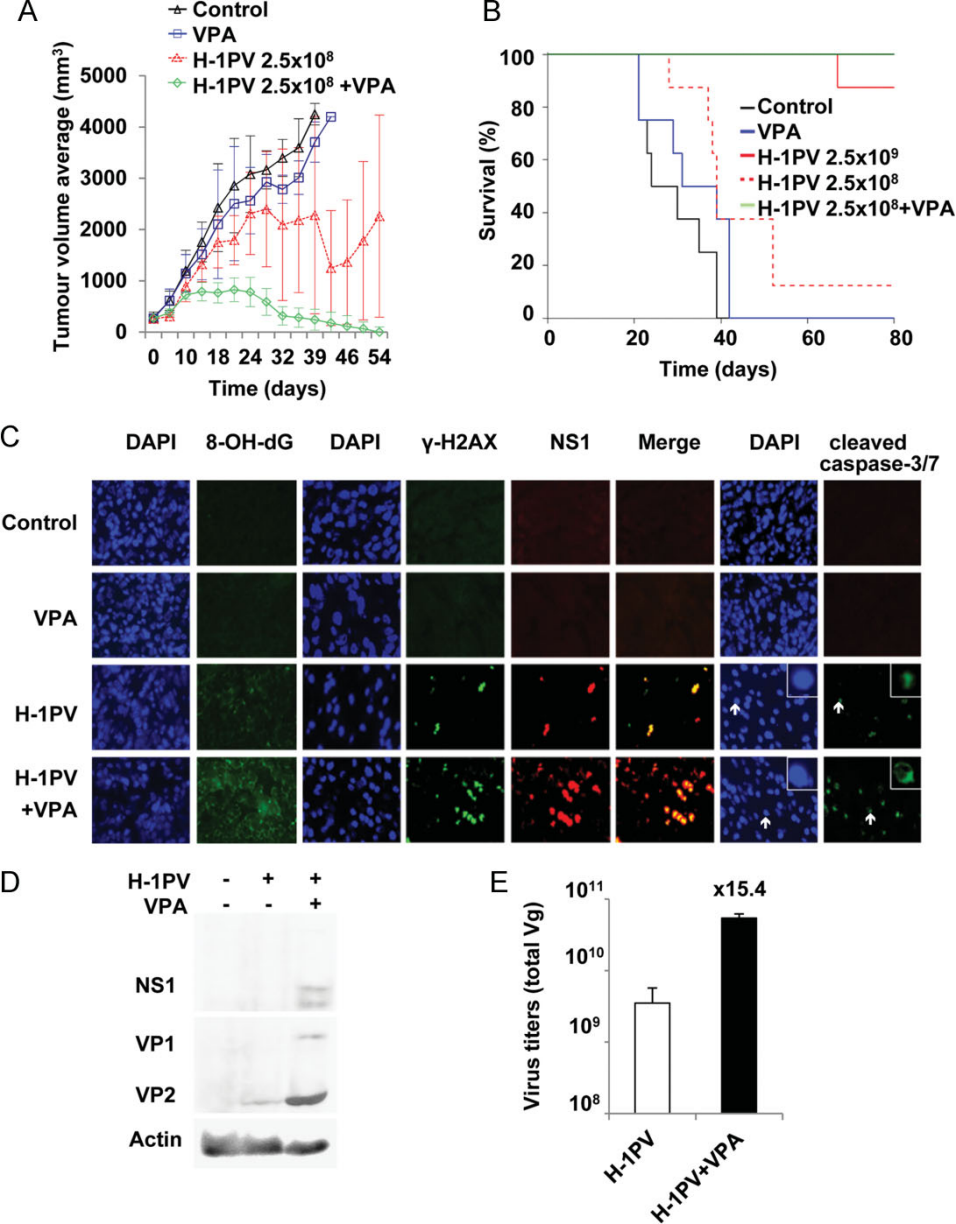
H-1PV/VPA co-treatment leads to complete eradication of established AsPC-1 tumours Source data is available for this figure in the Supporting Information. Tumour growth. Xenografts were established and animals treated as described in Materials and Methods Section. Average tumour values from eight animals are plotted with standard deviation bars.Survival curves. Survival of the tumour-bearing rats treated as indicated was analysed using the method of Kaplan–Meier. Statistically significant differences in survival were found in co-treated animals, as compared to animals treated with a single agent (*p* = 0.0032 as calculated with the two-sided log-rank test adjusted for multiple testing with the Bonferroni method). Other results from the statistical analysis are shown in Supporting Information Table S2).H-1PV/VPA co-treatment is associated with increased oxidative stress, DNA damage and apoptosis in treated tumours. Two rats of each group were sacrificed on day 27. Tumours were fixed, sectioned and examined by immunofluorescence staining with antibodies against 8-OH-dG (oxidative stress), γ-H2AX (DNA damage), NS1 (viral protein expression) and cleaved caspase-3/7 (apoptosis). Nuclei were visualized by DAPI staining.The viral proteins NS1, VP1 and VP2 accumulate to higher levels in tumour samples from VPA co-treated animals. Viral protein expression in tumour samples from H-1PV or H-1PV/VPA treated animals was measured as described in Fig 8D.VPA increases intratumoural H-1PV multiplication *in vivo*. Virus titers in tumour samples were determined as described in Fig 8E.

To test whether H-1PV and VPA also synergize *in vivo*, we used three different animal models: HeLa (CC) and AsPC-1(PDAC) xenografts in nude rats and PDAC tumour material from two patients xenotransplanted into NOD/SCID mice. When administered at high dosage (four fractional doses totalling 2.5 × 10^9^ pfu/animal, administered at 1 week intervals), H-1PV alone caused complete regression of tumours established from HeLa cells (Supporting Information Fig S7C). When the dosage was halved (1.25 × 10^9^ pfu/animal, fractionated as above) tumour growth slowed down but no regression was observed (Fig 8A and Supporting Information Fig S7D). When the dosage was further reduced (2.5 × 10^8^ pfu/animal), virus and mock treated animals showed no significant differences (Supporting Information Fig S7E and Table S2), indicating that a critical virus dose is required in order to achieve a therapeutic effect. On the other hand, VPA administrated at 100 mg/kg failed to impair tumour growth (Fig 8A and Supporting Information Fig S7B). Strikingly, tumour growth was reduced and then arrested in animals co-treated with low doses of H-1PV (1.25 × 10^8^ pfu/animal) and VPA (100 mg/kg; Fig 8A and Supporting Information Fig S7F–J). Most remarkably, this stabilization was followed by rapid regression leading to the complete disappearance of pre-existing tumours in all co-treated animals (Fig 8A and Supporting Information Fig S7). No loss of weight or other adverse side effect was documented in any of the treated animals (data not shown). In cured animals, NS1 was detected only in the kidneys at very low level, while being below the detection limit in all other organs (Supporting Information Fig S8). No tumour relapse was observed in co-treated animals over a 1-year follow-up (Fig 8B and data not shown). Morphological analysis of sections from H-1PV/VPA co-treated animals showed clear signs of tumour mass degradation, with large necrotic/apoptotic areas containing infiltrated macrophages and polynucleated giant cells most likely reabsorbing dead cells. Expression of the NS1 and VP viral proteins (Fig 8C and D) as well as virus multiplication (Fig 8E) were considerably higher in co-treated tumours. This was associated with a strong increase in the levels of γ-H2AX and cleaved caspase-3, indicating that tumour cells were undergoing apoptosis, most likely due to excessive DNA damage (Fig 8C). *In situ* hybridization assays performed with a probe specifically recognizing the DNA of human papillomavirus type 18 (which is integrated in HeLa cells), confirmed the massive disappearance of tumour cells in co-treated animals (Supporting Information Fig S9).A similar *in vivo* H-1PV/VPA synergy in tumour suppression was observed in animals grafted with the PDAC-derived AsPC-1 cell line. It is important to note that these cells are quite resistant to virus cytotoxicity *in vitro* (Fig 1A). Combined H-1PV and VPA treatment again led to complete regression of established tumours at virus doses that were ineffective when used as monotherapy (Fig 9A and B and Supporting Information Fig S10). Haematoxylin and eosin (H&E) staining of tumour specimens isolated from co-treated animals showed clear signs of tumour mass degradation, with large necrotic/apoptotic areas (Supporting Information Fig S11). Immunofluorescence analysis confirmed high NS1 protein levels, induction of oxidative stress, DNA damage and apoptosis in these sections (Fig 9C and D). As previously shown in HeLa xenografts, we also found that viral capsid protein steady levels as well as virus replication were enhanced in the presence of VPA (Fig 9D and E).

Finally, we tested the effectiveness of H-1PV/VPA co-treatment against fresh tumour material obtained after explorative laparotomy of two pancreatic cancer patients after single-passage expansion and grafting onto immunocompromised NOD-SCID mice. VPA treatment was found to slow down tumour growth but the effect remained incomplete in the xenografts of both patients. Tumours arising from Patient 1 material were very sensitive to H-1PV mono-therapy, with their growth being completely blocked, while Patient 2 xenografts proved less responsive to virotherapy. In contrast, H-1PV/VPA co-treatment was found to control all patient-derived neoplasias effectively (Supporting Information Fig S12). H&E-stained sections of tumours prepared at the end of the experiment revealed that necrotic areas were predominant in the H-1PV/VPA treated tumours (Supporting Information Fig S12). These results strongly support the combined use of H-1PV and VPA to treat CC and PDAC.

## DISCUSSION

Monotherapy of solid tumours often has limited success, as the tumour cells frequently develop resistance to the drug used. Investigators are therefore eager to find combinations of two or more therapeutics, acting through different mechanisms and exerting synergistic effects without increasing side effects. Preclinical studies revealed the antineoplastic potential of the rat parvovirus H-1PV and provided the basis of a phase I/IIa clinical trial using this agent as monotherapy in patients with glioblastoma multiforme (Geletneky et al, 2012). On the other hand, HDACIs also emerged recently as very promising anticancer agents (Rodriguez-Paredes & Esteller, 2011). Here we investigated the possibility of combining H-1PV with HDACIs and in particular with VPA, an HDACI commonly used as anti-epileptic drug, so as to reinforce their respective antineoplastic activities. We show that sub-lethal doses of VPA - compatible with its current clinical use (Reagan-Shaw et al, 2008) - significantly and synergistically increase H-1PV oncotoxicity both *in vitro* and *in vivo*, while the combination is not toxic for normal cells. Similarly, HDACIs have been shown to reinforce the cytotoxicity of other oncolytic viruses, including VSV, HSV (Katsura et al, 2009; Otsuki et al, 2008) and adenoviruses (VanOosten et al, 2006, 2007).

ROS have been reported to mediate the cytotoxicity of HDACIs (Minucci & Pelicci, 2006; Robert & Rassool, 2012). Although used at noncytotoxic doses in our experiments, VPA and NaB proved able to trigger ROS accumulation. On the other hand, we have previously shown that H-1PV infection is also associated with ROS accumulation in HeLa cells (Hristov et al, 2010). In the present study, we have confirmed the ability of the virus to induce ROS accumulation in other cell lines, supporting the concept that ROS are important mediators of viral cytotoxicity. Interestingly, H-1PV/VPA co-treatment results in a further increase in ROS generation. This suggests that the induction of oxidative stress contributes to the anticancer activity of the H-1PV/VPA combination, most likely by overwhelming the scavenging capacity of cellular antioxidative mechanisms. As ROS are a main cause of DNA damage (Kryston et al, 2011), their presence may also account for the increased DNA damage observed in tumour cells from co-treated cultures/animals, resulting in more effective activation of cell death pathways. An alternative, nonexclusive possibility would be that HDACIs facilitate parvovirus-triggered tumour cell death by lowering the threshold for induction of apoptosis. Indeed, HDACIs are also known to decrease levels of anti-apoptotic proteins (such as XIAP, c-FLIP, c-IAP2, survivin and Bcl-X_L_) and to increase levels of pro-apoptotic proteins (such as TRAIL, DR4 and DR5) (Matthews et al, 2012). Gene expression profiling studies have indicated that HDACI treatment dysregulates the transcription of about 10% of all cellular genes (Johnstone, 2002). Therefore it is conceivable that some of the genes affected by VPA treatment encode modulators of parvovirus replication and/or cytotoxicity. In particular, HDACIs can repress the expression of genes involved in antiviral innate immune response, thereby improving VSV and HSV replication and spread in cancer cells (Nguyen et al, 2010). However, the dependence of H-1PV/HDACI synergistic oncolysis on the acetylated status of NS1 argues against the hypothesis that VPA-mediated down-regulation of the cellular antiviral response plays a central role in enhancing H-1PV tumour suppression. VPA treatment may thus enhance H-1PV viral oncolysis by multiple mechanisms. The present study shows that in H-1PV-infected tumour cells, the NS1 protein gets acetylated at two major sites and that the fraction of acetylated NS1 increases upon VPA treatment resulting in enhanced NS1-dependent gene expression, virus replication and cytotoxicity. In keeping with the role of acetylation in the functional activation of NS1, simultaneous mutation at both acetylation sites almost completely abolished NS1 transcriptional and cytotoxic activities and strongly impaired virus replication and cytotoxicity (while single mutant had an intermediate phenotype). Remarkably, VPA-dependent stimulation of NS1 functions in permissive HeLa cells resulted in enhanced viral multiplication and release both *in vitro* and *in vivo*. A lower but substantial increase in virus yields, was also achieved by the H-1PV/VPA combination in SiHa and CaSki cell cultures and in AsPC-1 xenografts. Thus, acetylation appears to boost H-1PV oncotoxicity at both qualitative and quantitative levels, by stimulating NS1 intrinsic toxic activity and virus production, respectively. These results are of clinical interest as they may allow the use of lower viral doses to reach therapeutic efficacy. Although the primary aim of the present study was not to elucidate the role of acetylation in NS1 functioning, our results are bound to raise interest in this question and to open new lines of research. As VPA treatment increased the capacity of NS1 to bind to the viral P38 promoter, we propose that the enhanced NS1 transcriptional activity observed in the presence of VPA is due to the greater affinity of the acetylated protein for its cognate DNA sequences. It is still possible that, together with phosphorylation (Nuesch et al, 2012), acetylation may also modulate NS1 homo-dimerization, interaction with cell proteins, subcellular localization and/or stability. VPA treatment enhances the ability of NS1 to trans-activate the P38 promoter, resulting in higher levels of VP1 and VP2 capsid proteins. Additional experiments are required to determine whether VPA may also affect the capacity of NS1 to regulate its own P4 promoter.

We have identified K85 and K257 as acetylation sites of NS1. Sequence alignment revealed that the two K residues are conserved in most members of the *Parvoviridae* family. In particular K85 is shared with minute virus of mice (MVM), LuIII, Canine parvovirus (CPV), Feline panleukopenia virus (FPV) and Kilham rat virus (KRV), while K257 is shared with CPV, FPV, Porcine parvovirus, KRV, Mink enteritis virus and Rat minute virus (data not shown). This conservation suggests that acetylation may also play an important role in modulating the activities of other parvoviruses NS1 proteins. Interestingly, K85 is located in the NS1 DNA-binding domain and K257 lies near the NS1 dimerization domain (aa 258–278). These domains are both essential to the transcriptional and cytotoxic activities of the related Minute Virus of Mice (MVM) NS1 protein (Nuesch, 2006). It should also be stated that other acetylation site(s) besides K85 and K257 may exist in the NS1 protein as NS1 acetylation is strongly decreased but not completely abolished when both K85 and K257 were mutated. Our MS analysis covered 74.2% of the NS1 amino-acid sequence (499 out of 672 aa). The remaining part includes 12 lysines (out of 48 residues) that could theoretically be acetylated. Yet, the strong reduction in NS1 acetylation observed after mutagenesis of above residues, argues against the existence of many other major acetylation sites within the NS1 sequence.

The histone acetyl transferase(s) [HAT(s)] and HDAC(s) involved in NS1 acetylation/deacetylation remain to be identified. As acetylation regulates the activity of both histones and nonhistone proteins (Lane & Chabner, 2009; Spange et al, 2009), it is tempting to speculate that by interacting with its cellular (de)acetylators the virus may modulate acetylation signalling in the host cells and modify the cellular environment to successfully accomplish its life cycle. For instance, NS1 might compete with cellular HAT/HDAC substrates and/or sequester distinct HATs/HDACs away from their normal targets and/or even redirect these enzymes to cellular substrates controlling the virus life cycle. Other viruses, in particular HIV and adenoviruses, were reported to disrupt the normal functions of HATs and HDACs and manipulate in this way essential cell components (*e.g*. transcriptional machinery) in order to activate viral gene expression and replication (Caron et al, 2003). It is notable that NS1 mediates the general dysregulation of host gene expression (Li et al, 2005a) occurring upon H-1PV infection (our unpublished results). Interestingly, expression of a gene (*ciliary neurotrophic factor receptor alpha*) associated with virus-mediated tumour reversion was found to be up-regulated by NS1 of the related rat parvovirus RPV/UT through histone acetylation (Iseki et al, 2005).

**Figure 10:**
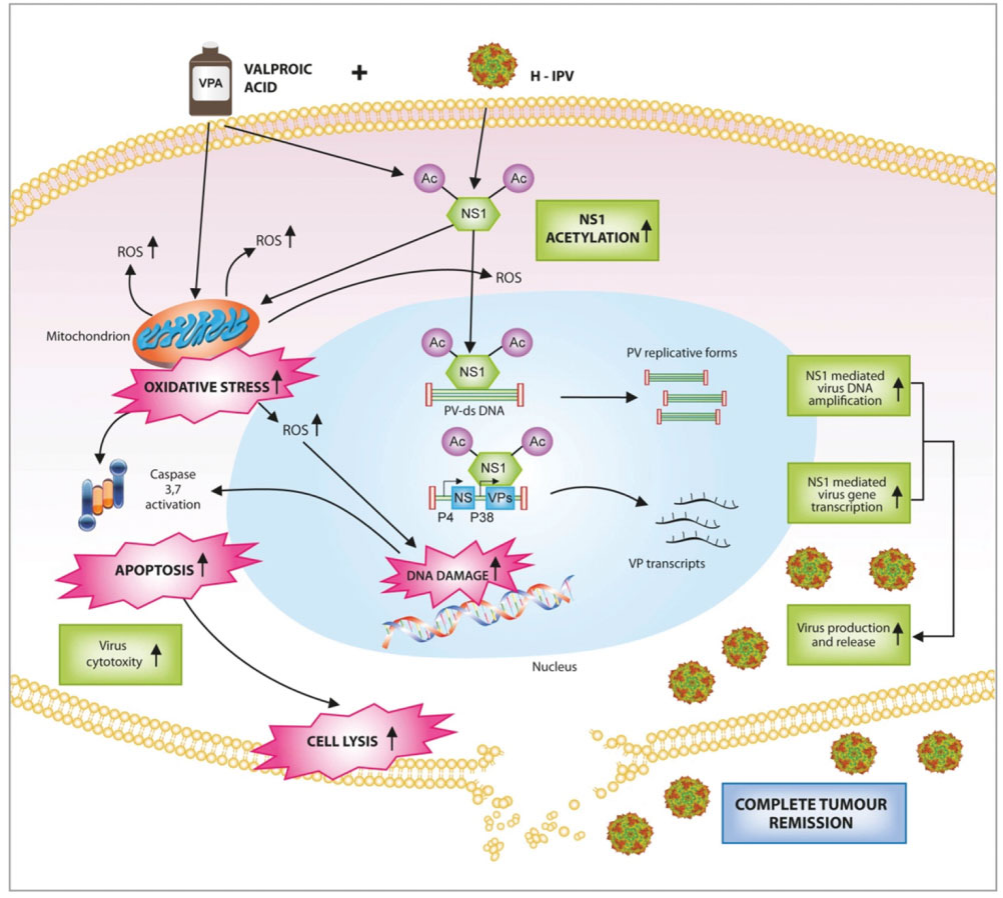
H-1PV and VPA synergize against cancer: a tentative model Multiple mechanisms of action are thought to play a role in the enhanced anticancer effect observed by combining H-1PV with VPA. On the one hand, VPA and H-1PV (through NS1) induce accumulation of intracellular reactive oxygen species (ROS). This oxidative stress overwhelms antioxidant mechanisms leading to increased DNA damage, apoptosis and cell lysis. On the other hand, VPA treatment is associated with hyper-acetylation of the viral NS1 protein which is converted in a more active peptide. Acetylated NS1 binds more efficiently to viral DNA, leading to increased viral DNA replication and viral gene transcription. This ultimately results in enhanced viral multiplication in permissive cancer cells. Viral progeny particles infect neighbouring cancer cells and participate in the complete tumour remission observed in animal models. An additional component not documented by the present study, may involve the VPA-mediated modulation of cellular defence mechanisms (not shown in scheme). Abbr. NS1: parvovirus nonstructural NS1 protein; PV-ds DNA: H-1PV double stranded DNA replicative form; P4: H-1PV early promoter; P38: H-1PV late promoter; NS: transcription unit encoding the nonstructural NS proteins; VP: transcription unit encoding the VP capsid proteins; VPA: valproic acid.

In conclusion, this study shows that the therapeutic potential of H-1PV can be strikingly increased by VPA. This combination treatment provides a complete and persistent regression of engrafted human carcinomas under conditions in which the virus alone was not fully effective even at 20-fold higher dose. Importantly, the synergistic antitumour effect of H-1PV/VPA co-treatment is achieved under conditions in which the safety profile of both of its components is preserved. We discovered a new regulation of parvoviral protein NS1 whose intrinsic oncotoxicity and capacity for driving virus multiplication are both stimulated through VPA-enhanced acetylation of specific K residues (Fig 10). The present proof-of-concept study gives efficacy and safety arguments warranting the further clinical evaluation of the H-1PV/VPA combination for use against life-threatening cancers.

## MATERIALS AND METHODS

### Generation of plasmids

NS1 mutants in which the two identified acetylation sites (lysines 85 and 257) are mutated singly or together to prevent acetylation (substitution with arginine) were generated with the QuickChange Multi Site Directed Mutagenesis Kit (Stratagene, Heidelberg, Germany) according to the manufacturer's instructions. A construct corresponding to BSK-Flag-haemagglutinin (HA)-tagged wild-type NS1 was used as template for the mutagenesis reaction. It was obtained by subcloning the Flag-HA-NS1 gene of pCDNA5/TO-Flag-HA-NS1-H1 (Hristov et al, 2010) into the *EcoR*I/*Not*I digested pBluescript SK vector (Promega, Mannheim, Germany). The following primers were designed to introduce the mutation: K85R For: 5′-GTG GAT GAC ATG ACC AAA A**G**G CAA GTA TTT ATT TTT GAT TC-3′; K85R Rev 5′-GA ATC AAA AAT AAA TAC TTG C**C**T TTT GGT CAT GTC ATC CAC-3′; K257R For 5′-CTG TAT ACT GAT GAG ATG A**G**A CCA GAA ACG GTC GAG ACC-3′; K257R Rev 5′-GGT CTC GAC CGT TTC TGG T**C**T CAT CTC ATC AGT ATA CAG-3′ (the mutations introduced are in boldface). After mutagenesis, the NS1 mutants were subcloned into the pCDNA4/TO plasmid (Invitrogen, Life Technologies, Darmstadt, Germany) using the *EcoR*I-*Not*I restriction sites, thus generating pCDNA4/TO-NS1-K85R, pCDNA4/TO-NS1-K257R and pCDNA4/TO-NS1-K85R-K257R.

To generate acetylation-defective NS1 mutant viruses (H-1PV-K85R, H-1PV-K257R and H-1PV-K85R-K257R), a fragment of the H-1PV genome containing the NS transcription unit, obtained by digesting the pSR19 clone (Kestler et al, 1999) with *Sal*I-*EcoR*I, was first cloned into the pBluescript SK + plasmid (Stratagene). This construct was then used as template for the *in situ* mutagenesis reaction using the primers described above. The modified fragments were then cloned back into their parental pSR19 backbone to generate the pH-1PV-K85R, pH-1PV-K257R and pH-1PV-K85R-K257R infectious clones. The pGL3-H-1PV-P38 plasmid, containing the H-1PV P38 late promoter in front of the (*firefly*) luciferase reporter gene was constructed by inserting the *Nhe*I-*Hind*III fragment extracted from pSR19 into the similarly digested pGL3 basic vector (Promega). All the generated plasmids were DNA sequence-verified.

### Establishment of stable cell lines

Generation of the HeLa-NS1 stable cell line expressing Flag-HA-tagged H-1PV NS1 under a dox-inducible promoter has been previously described (Hristov et al, 2010). The HeLa-NS1-K85R, HeLa-NS1-K257R and HeLa-NS1-K85R-K257R stable cell lines (all the NS1 mutants are Flag-HA tagged) were generated by transfecting HeLa T-Rex cells (Invitrogen) with the pCDNA4/TO-NS1-K85R or pCDNA4/TO-NS1-K257R or pCDNA4/TO-NS1-K85R-K257R plasmid. Selection of positive clones was done on a medium containing 6 µg/ml blasticidin and 200 µg/ml zeocin, according to the manufacturer's protocol. A pool of selected clones was used in this study. NS1 expression was induced by adding 1 µg/ml dox.

### Cells

The cervical carcinoma-derived cell line CaSki and the pancreatic carcinoma-derived lines T3M-4, MiaPaCa-2 and AsPC-1 were purchased from ATCC (LGC Standards GmbH, Wesel, Germany). The HeLa and SiHa cervical carcinoma cell lines were a gift from A. Alonso (German Cancer Research Center, Heidelberg, Germany). The early-passage cervical-carcinoma-derived cell cultures (CxCa) positive for human papillomavirus 16 were a gift from Andreas M. Kaufmann (Charité-Universitätsmedizin Berlin, Germany). Human primary oral and foreskin fibroblasts were a gift from Massimo Tommasino (IARC, Lyon, France). Human primary adult melanocytes were from Invitrogen (Carlsbad, CA). Human astrocytes were obtained from ScienCell Research Laboratories (San Diego, CA). Cell lines were authenticated using Multiplex Cell Authentication by Multiplexion (Heidelberg, Germany) as described recently (Castro et al, 2013).

The HeLa, CaSki, SiHa, CxCa, human foreskin fibroblast and AsPC-1 cell lines were grown in Dulbecco's modified Eagle's medium (DMEM, Sigma–Aldrich, Munich, Germany) supplemented with 10% fetal bovine serum (FBS, Gibco, Life Technologies, Darmstadt, Germany). The medium for CxCa cells additionally contained 25 mM Hepes (Gibco). T3M-4 and MiaPaCa-2 were cultured in Roswell Park Memorial Institute medium 1640 (RPMI, Invitrogen) supplemented with 10% FBS. All media contained 2 mM l-glutamine (Gibco), 100 U/ml penicillin and 100 µg/ml streptomycin (Gibco). HeLa-NS1 stable cell lines were maintained in tetracycline-free DMEM supplemented with 6 µg/ml blasticidin and 200 µg/ml zeocin (Invitrogen). Human primary oral fibroblasts were maintained in DMEM (Gibco) without l-glutamine or phenol red. Human astrocytes were cultured in astrocyte medium (Gibco). Human melanocytes were grown in medium 254 supplemented with human melanocyte growth supplement (HMGS, Invitrogen). Cells were grown at 37°C, 5% CO_2_ and 95% humidity and routinely checked for mycoplasma contamination.

### HDAC inhibitors

Sodium butyrate and VPA were purchased from Sigma–Aldrich and Alexis Biochemicals (Enzo Life Science, Lörrach, Germany), respectively. The dose of VPA used in animal experiments was 100 mg/kg. This dose corresponds to the human equivalent of 16 mg/kg, according to the body surface area normalization method for conversion of drug doses between species as recommended by the Food and Drug Administration (Reagan-Shaw et al, 2008). This dose is within the clinical range of 15–30 mg/kg used for long-term treatment of epileptic patients and is far below the limit of 60 mg/kg considered safe and well tolerated in humans (Atmaca et al, 2007).

### Virus production and titration

Wild-type and mutant H-1PV viruses were produced, purified and titrated as previously described (Allaume et al, 2012).

### Lactate dehydrogenase (LDH) assay

Cells were seeded into a 96-well plate at the density of 2000 cells/well for HeLa, CaSki, SiHa, CxCa and AsPC-1 cells, 4000 cells/well for T3M-4, MiaPaCa-2 and human primary normal cells (POF, HFF, melanocytes and astrocytes), and 6000 cells/well for the HeLa-NS1 stable cell line in 50 µl of their respective culture media supplemented with 10% heat-inactivated FBS. After 24 h, 50 µl FBS-free medium with or without H-1PV and/or VPA was added to the cells, with the exception of HeLa-NS1 cells, in which case the medium added contained dox (or not) and VPA (or not). For each condition tested, seven replicates were prepared of which three were used for calculating the total cell lysis in the presence of detergent. After 72 h, the cells were processed for the LDH assay (CytoTox 96 nonradioactive cytotoxicity assay, Promega) as previously described (El-Andaloussi et al, 2012).

### ROS content

Cancer cell lines were plated onto 6-well plates or 10-cm dishes and infected or not with H-1PV in the presence or absence of VPA (1 mM for all cell lines except for the T3M-4 cells, in which the drug was used at 0.5 mM). The following viral MOIs were used: HeLa, 1 pfu/cell; SiHa and MiaPaCa-2, 5 pfu/cell; CaSki, 10 pfu/cell; CxCa, 25 pfu/cell; T3M-4, 20 pfu/cell; AsPC-1, 50 pfu/cell. HeLa-NS1 cells were treated (NS1-induction) or not with dox with or without 1 mM VPA. 24–48 h post-treatment, the cells were loaded with 10 µM dichlorofluorescein diacetate (DCFH-DA, Sigma–Aldrich) for 1 h at 37°C. In the presence of ROS, DCFH-DA is oxidized and converted to a highly fluorescent product (Armstrong & Whiteman, 2007). After trypsinization, the cells were washed twice with phosphate-buffered saline (PBS), resuspended in 500 µl PBS and directly analysed by flow cytometry (FACSort, Becton Dickinson). At least 20,000 events were acquired and analysed with the CellQuest software (Becton Dickinson).

### Protein extraction and Western blot analysis

Cells were scraped in the culture medium, harvested and washed with PBS. Cell pellets were lysed on ice for 30 min in lysis buffer (20 mM Tris–HCl at pH 7.5, 1 mM EDTA, 150 mM NaCl, 0.1% SDS, 1% Triton X-100, 1% Na-deoxycholate) containing protease (Roche Diagnostics, Mannheim, Germany) and phosphatase inhibitors (Sigma–Aldrich). Cell debris was removed by centrifugation at 13,000 rpm for 15 min at 4°C. Cell lysates from tumour xenografts were prepared as previously described (Verollet, 2008). Briefly, rats were euthanized by inhalation of gaseous carbon dioxide (CO_2_), tumours were excised and snap frozen in liquid nitrogen. About 50 mg of tumour sample were transferred into 2 ml ceramic beads-filled tubes (Precellys, PEQLAB Biotechnologie GmbH, Erlangen, Germany) in the presence of 300 µl of lysis buffer (20 mM Tris–HCl, pH 7.5, 1 mM EDTA, 1 mM EGTA, 150 mM NaCl, 0.5% NP-40, 0.5% sodium deoxycholate, 10 mM NaF, 10% glycerol, 1 mM PMSF, 10 mM nicotinamide, 10 mM sodium butyrate and protease inhibitor cocktail). After Precellys®24 homogenization or vortexing, samples were centrifuged at 12,000 rpm for 10 min at 4°C. The supernatants were kept at −80°C for further analysis.

Total cell extracts (20 µg) were resolved by sodium dodecyl sulphate–polyacrylamide gel electrophoresis (SDS–PAGE) and transferred onto a Hybond-P membrane (GE Healthcare). The following antibodies were used for analysis: mouse monoclonal anti-actin (clone C4, MP Biomedicals, Illkirch, France; used at 1:10,000 dilution), polyclonal anti-NS1 SP8 antiserum (Bodendorf et al, 1999) (1:3000), polyclonal anti-VP2 antiserum (1:2000; Kestler et al, 1999), phospho-Ser139 histone H2AX (γ-H2AX; Abcam PLC, Cambridge, UK; 1:1000).

### Real-time detection of cell proliferation

Cells were seeded into a 96-well E-plate (Roche Diagnostics) at the density of 8000 (HeLa) or 16,000 cells/well (HeLa-NS1 stable cell line). After 24 h, the cells were either infected with H-1PV (HeLa) or induced with dox (HeLa-NS1) and grown in the presence or absence of VPA. Cell proliferation and growth were measured in real time every 30 min with the xCELLigence system (Roche Diagnostics) according to the manufacturer's instructions and expressed as normalized cell indexes. Each growth condition was analysed in quadruplicate. Average values of each experimental condition are presented with the relative standard deviation.

### Detection of apoptosis

HeLa-NS1 and HeLa cells were plated onto coverslips and grown overnight before being treated or not with dox (HeLa-NS1) or H-1PV (HeLa) in the presence or absence of VPA (1 mM). 24 h post-treatment, the cells were loaded with 5 µM CellEvent™ Caspase-3/7 Green Detection Reagent (Invitrogen) for 30 min, followed by 4',6-diamidino-2-phenylindole (DAPI) staining. Positive cells were analysed on a fluorescence microscope (KEYENCE BZ-9000E, Japan) with the BZ-II Analyzer software.

### Immunoprecipitation

H-1PV-infected HeLa cells were grown in 15-cm dishes at 70% confluency in the presence or absence of VPA (1 mM). After 16 or 32 h of incubation, cells were washed twice with PBS and harvested using a plastic cell lifter. Cell pellets were lysed in IP buffer (20 mM Tris–HCl, pH 7.5, 1 mM EDTA, 1 mM EGTA, 150 mM NaCl, 0.5% NP-40, 0.5% sodium deoxycholate, 10 mM NaF, 10% glycerol, 1 mM PMSF, 10 mM nicotinamide, 10 mM sodium butyrate and protease inhibitor cocktail). Total cell lysates (2 mg) were pre-cleared with 20 µl protein A plus G agarose beads for 2 h and then incubated overnight with the polyclonal anti-NS1 SP8 antiserum (Hristov et al, 2010), after which 20 µl protein A plus G agarose beads were added to the mixture. After incubation for an additional 4 h, the beads were extensively washed with IP buffer. Immunoprecipitated proteins were directly eluted from the beads by boiling the samples with SDS protein sample buffer and resolved by SDS–PAGE. An antibody against acetylated lysine (clone 9441, Cell Signaling, Technology, Inc., Danvers, MA) was used to detect acetylated NS1. The membrane was stripped and re-probed with monoclonal anti-NS1 3D9 antiserum (Hristov et al, 2010) for detection of total NS1 protein.

For the immunoprecipitation of Flag-tagged NS1, the HeLa-NS1, HeLa-NS1-K85R, HeLa-NS1-K257R and HeLa-NS1-K85R-K257R stable cell lines were grown in 15-cm dishes in a medium supplemented or not with dox and/or VPA and lysed with lysis buffer (50 mM Tris–HCl, pH 7.9, 137 mM NaCl, 10 mM NaF, 1 mM EDTA, 1% Triton X-100, 0.2% sarkosyl, 10% glycerol, 1 mM PMSF, 10 mM nicotinamide, 10 mM sodium butyrate acid and protease inhibitor cocktail). The whole-cell extracts were subjected to immunoprecipitation with the monoclonal anti-Flag M2-agarose-bead-conjugated antibody (Sigma–Aldrich). Immunoprecipitated proteins were eluted and analysed as described above.

### Luciferase reporter assay

HeLa cells were grown in a 12-well plate. Lipofectamine™ 2000 (Invitrogen) was then used to transfect the cells with a DNA mixture composed of 800 ng pGL3-H-1PV-P38 (containing the H-1PV P38 promoter in front of the firefly luciferase reporter gene), 150 ng pRL-TK plasmid (Promega) (expressing *Renilla* luciferase, used to normalize the transfection efficiency) and 800 ng pCDNA4/TO expressing NS1 wild-type or an acetylation-defective NS1 mutant (NS1-K85R, NS1-K257R or NS1-K85R-K257R). After 24 h, the transfected cells were split into a 96-well plate, and grown for one additional day. For the experiment shown in Fig 3B, transfected cells were grown in a medium supplemented or not with VPA (1 mM).

For the experiment shown in Supporting Information Fig S5, HeLa-NS1 cells were seeded into 10-cm dish (2 × 10^6^ cells/dish) and cultured for 24 h. Cells were then transfected with 4 µg of pGL3-H-1PV-P38 or pGL3 basic vector (Promega) by using the FuGENE HD transfection reagent (Roche) in a 1:3 ratio (µg DNA:µl FuGENE). The next day, transfected cells were seeded in 12-well plates, cultivated for one additional day and then treated with doxycycline (1 µg/ml) with or without NaB (1 mM) for 48 h. Cells were then incubated with 50 µl of 1× passive lysis buffer and luciferase activity was measured in triplicate for each experimental condition using the Dual Luciferase Assay System (Promega) according to the manufacturer's instructions. Luciferase values shown in Supporting Information Fig S5 were normalized for protein concentration, as determined by the bicinchoninic acid (BCA) assay (Thermo Fisher Scientific) according to the manufacturer's instructions.

### RNA isolation and real-time quantitative RT-PCR

Total RNA was isolated using the TRIzol® Reagent RNA-extraction kit (Invitrogen) according to the manufacturer's instructions. 1 µg total cellular RNA was digested with 1 unit DNase I (Promega) at 37°C for 20 min to remove genomic DNA contamination, before processing for reverse transcription (RT) with oligo(dT) primers and reverse transcriptase of Moloney murine leukaemia virus (Promega). For each cDNA sample, a control was produced with an RT mixture to which no reverse transcriptase was added, in order to detect potential contamination of the cDNA sample with residual genomic DNA. Quantitative PCR using a fraction of the cDNA as a template was performed with the following primer pairs: EGFP-for 5'-ATCATGGCCGACAAGCAGAAGAAC-3' and EGFP-rev 5'-GTACAGCTCGTCCATGCCGAGAGT-3'; H-1PV-NS1-for 5'- GCGCGGCAGAATTCAAACT-3' and H-1PV-NS1-rev 5'-CCACCTGGTTGAGCCATCAT-3'; GAPDH-for 5'-ATGACATCAAGAAGGTGGTC-3' and GAPDH-rev 5'-CATACCAGGAAATGAGCTTG-3'. *GAPDH* was chosen as internal control among 19 human reference genes (RealTime *ready* Human Reference Gene Panel, Roche Diagnostic) because its expression was stable after virus infection (data not shown). The threshold cycle of fluorescence (Ct) for each sample was determined by real-time PCR using the Mastercycler® ep realplex system (Eppendorf, Hamburg, Germany). Relative quantification of gene expression between the groups was performed applying the 2-ΔΔCt method (Livak & Schmittgen, 2001).

### Chromatin immunoprecipitation and PCR amplification

HeLa cells grown in 10-cm dishes (2 × 10^6^ cells/dish) were infected with H-1PV wild-type or the H-1PV-NS1-K85R-K257R mutant (MOI 0.5 pfu/cell) and grown in the presence or absence of VPA (1 mM) for 24 h. Cellular DNA/protein complexes were cross-linked with 1% formaldehyde and cell lysates prepared by sonication as previously described(Sandmann et al, 2006). Sheared cross-linked chromatin (40 µg) was incubated with NS1 SP8 or control IgG antibodies at 4°C overnight in 800 µl of 16.7 mM Tris–HCl (pH 8.0), 167 mM NaCl, 0.01% SDS, 0.1% Triton X-100 containing buffer. Immunoprecipitates were captured on protein A/G–Sepharose pre-saturated for 2 h with salmon sperm DNA (0.25 mg/ml). Protein-DNA complexes were washed twice in low-salt buffer [150 mM NaCl, 50 mM Tris–HCl (pH 8.0), 5 mM MgCl2, 1% Triton], twice in the same buffer containing 500 mM NaCl, once in a buffer containing 250 mM LiCl, 10 mM Tris–HCl (pH 8.0), 5 mM EDTA, 0.5% Na-deoxycholate, 0.5% Triton and twice in Tris–EDTA (TE) buffer. After elution, reversion of the cross-links (6 h at 65°C) and digestion with proteinase K, the precipitated DNA was extracted and analysed by quantitative RT-PCR with an Abi Prism 7300HT sequence detection system (Applied Biosystems, Life Technologies, Darmstadt, Germany). The following primers were used: P38-ChIP-For 5'-TCAAAGCTATTTGTTCTGGCCA-3', and P38-ChIP-Rev 5'-CAAACCAAAGTCACCAGGTAGT-3' (amplification of the 1621–1833 region of the H-1PV genome including the P38 promoter). To check the specificity of the DNA co-immunoprecipitation, the following primers were used: VP end-H-1PV-For 5'-AAAACA ACCCACCAGGTCAA-3' and VP end H-1PV-Rev 5'-ATTGGCAACAGAGTCTGTGGTT-3' (amplification of the 4274–4474 region of the H-1PV genome outside the P38 promoter). Non-immunoprecipitated chromatin was used as total input control. Results were expressed as Ct values and measured in duplicates for each experimental condition.

### DNA extraction and Southern blotting

Cells were harvested in extraction buffer [10 mM Tris–HCl (pH 7.4), 10 mM EDTA, 0.6% SDS], and digested with proteinase K (100 mg/ml) for 3 h at 37°C. Total DNA was sheared by several passages through a 0.5 µm needle and analysed by agarose gel electrophoresis. After in-gel DNA denaturation and neutralization, the DNA was transferred onto a nitrocellulose membrane and UV cross-linked to the membrane. Viral DNA was detected with a ^32^P-labeled probe corresponding to the *EcoR*V (nt 385)-*EcoR*I (nt 1885) fragment of the pMVM plasmid.

### Parvovirus replication and production: real-time qPCR and plaque assay

HeLa cells (4 × 10^4^ cells/well) were seeded in 12-well plates. After 24 h, cells were infected with H-1PV at a MOI of 0.01 pfu/cell, in the presence or absence of VPA (1 mM). After further incubation for 24–120 h, cells were harvested by scraping in their medium and subjected to three freeze-thaw cycles. Crude cell extracts were digested with 50 U/ml of benzonase® Nuclease [Ultrapure grade, (Sigma–Aldrich Chemie GmbH, Steinheim, Germany)] for 30 min at 37 °C to remove non-encapsidated viral DNA. Viral genomic DNA was purified from cell lysates by using the QiaAmp MinElute virus kit (Qiagen, Hilden, Germany) according to the manufacturer's instructions. Quantification of viral DNA was carried out by real-time qPCR, as previously described (El-Andaloussi et al, 2012). To assess virus replication *in vivo*, 50 µl of the cell lysate obtained from tumour xenografts were digested with 50 U/ml of benzonase® Nuclease and subjected to virus DNA purification as described above.

For the experiment shown in Supporting Information Fig S6, 1 × 10^6^ of SiHa or Caski cells were seeded into 10-cm dishes. On the next day, cells were infected with H-1PV at MOIs of 0.2 and 1 pfu/cell (SiHa) or MOI of 1 and 5 pfu/cell (Caski) in the presence or absence of VPA (1 mM). After 96 h, cells were harvested by scraping in their medium and lysed by three freeze-thaw cycles. Parvovirus titers of the crude extracts were determined by plaque assay as previously described (El-Andaloussi et al, 2012).

### Mass spectrometry

To purify the acetylated NS1 protein, 5 × 10^6^ HeLa-NS1 cells were grown for 24 h in a medium supplemented with dox (in order to induce NS1 expression) and VPA (1 mM) or NaB (1 mM) plus 5 mM nicotinamide (Sigma–Aldrich; this last was added 6 h before harvest). The cells were lysed in Flag-lysis buffer consisting of 50 mM Tris–HCl (pH 7.9), 137 mM NaCl, 10 mM NaF, 1 mM EDTA, 1% Triton X-100, 0.2% sarkosyl, 10% glycerol and fresh protease inhibitor cocktail (Sigma–Aldrich) supplemented with 2 mM TSA and 10 mM nicotinamide (Tang et al, 2008). The NS1 protein was immunoprecipitated with anti-Flag M2 monoclonal antibody conjugated to agarose beads (Sigma–Aldrich). The eluted material was resolved by 2D SDS–PAGE. Following Coomassie staining, bands corresponding to the NS1 protein were excised from the gel and proteins in the gel pieces were reduced with 10 mM DL-Dithiothreitol (DTT) for 1 h at 56°C and alkylated with 55 mM iodoacetamide for 30 min at room temperature. The gel pieces were washed three times alternately with H_2_O and H_2_O/acetonitrile (ACN) (50:50, v/v) and finally with 100% ACN. Proteins were in-gel digested with trypsin at 37°C overnight. Tryptic peptides were extracted from the gel pieces with acetonitrile/0.1% trifluoroacetic acid and analysed by nanoLC-ESI-MS/MS. Peptides were separated with a nanoAcquity UPLC system. Peptides were loaded onto a C18 trap column (180 µm × 20 mm) with a particle size of 5 µm (Waters GmbH, Eschborn, Germany). Liquid chromatography separation was performed on a BEH130 C18 main column (100 µm × 100 mm) with a particle size of 1.7 µm (Waters GmbH, Eschborn, Germany) at a flow rate of 0.4 µl/min. The eluent was a 1-h gradient from 0% to 90% solvent B in solvent A. Solvent A contained 98.9% water, 1% ACN, 0.1% formic acid and solvent B contained 99.9% ACN and 0.1% formic acid. The nanoAcquity UPLC system was coupled online to an LTQ Orbitrap XL mass spectrometer (Thermo Scientific, Bremen, Germany). Data were acquired by scan cycles of one FTMS scan with a resolution of 60,000 at a m/z ratio of 400 and a range from 300 to 2000 m/z in parallel with six MS/MS scans in the ion trap of the most abundant precursor ions. Instrument control, data acquisition and peak integration were performed with the Xcalibur software 2.1 (Thermo Scientific).

Database searches were performed against the NCBInr database (release 120608) with the MASCOT search engine (Matrix Science, London, UK; version 2.1). The selected taxonomy was viruses. The peptide mass tolerance for database searches was set at 6 ppm, and fragment mass tolerance at 0.7 D. The statistical significance threshold was set to *p* < 0.05. Carbamidomethylation of cysteine residues was set as fixed modification. Variable modifications included: oxidation of methionine, acetylation of lysine, phosphorylation of serine, threonine, and tyrosine, and methylation of lysine, histidine, and arginine. One missed cleavage site in case of incomplete trypsin hydrolysis was allowed.

### Animal studies

#### HeLa and AsPC-1 xenografts

Five- to six-week-old nude female rats (Charles River) were housed in isolated laminar flow hoods at the Animal Facility of the German Cancer Research Center in Heidelberg. Housing and treatment of animals were in accordance with institutional and state guidelines and approved by the internal Ethics Committee. The nude rats were subcutaneously implanted with 5 × 10^6^ cancer cells in the right hind flank. Nodules reached a volume of 250–450 mm^3^ in about 1 week of time. On day 0, the animals were randomized into groups (*n* = 8) and treated with PBS (negative control) or VPA (100 mg/kg/day; administered intraperitoneally) or with different doses of H-1PV (injected intratumourally, 2/5 of the total viral dose indicated in Supporting Information Fig S7 and S10) or with a combination of both agents. Virus treatment was repeated three more times (on days 7, 14 and 21), but with half of the dose administered on day 0. VPA was given daily (Monday to Friday) for the first 28 days. Tumour diameters were measured with a digital caliper every other day, and the tumour volume in mm^3^ was calculated by the formula: Volume = width^2^ × length/2(Balko et al, 2012). Animal weight was measured once a week. Rats were sacrificed when the tumour mass reached 4000 mm^3^, in keeping with animal welfare regulations.

For the *in vivo* assessment of viral protein expression and replication, shown in Fig 8 and 9 (panels D and E), animals were randomized at day 0 into two groups (*n* = 3) and treated by intratumoural injection of 2.4 × 10^9^ H-1PV virus genome (Vg)/animal with or without intraperitoneal administration of VPA (100 mg/kg/d). Virus treatment was repeated one more time (on day 7), but with half of the dose administered on day 0. VPA was given daily (Monday to Friday) for the 14 days. Tumours were collected and processed for the measurement of virus protein expression and production as described above.

#### Xenotransplantation of patient material

Six-week-old NOD/SCID mice (C.B.-17/IcrHanHsd-Prkdc-scid) were purchased from Harlan (Gannat, France), kept in filter cages and fed sterile chow and water *ad libitum*. Operational tumour material was obtained during explorative laparotomies from patients of the University Clinic Heidelberg, and pathologically confirmed as pancreatic ductal carcinoma (Ethic Votum Nr. 301/2001). Material was cut with a sterile scalpel into 1–2-mm pieces in PBS in a 10-cm^2^ Petri dish. Pieces were cryopreserved in 60% RPMI 1640, 30% FCS, 10% DMSO medium in liquid nitrogen tank until used. For implantation, the pieces of material were thawed and washed three times with RPMI. The mice were anaesthetized during the entire manipulation time by continuous inhalation of an isofluran + O_2_ mixture. Two 1–2-mm pieces per animal were implanted in subcutaneous cavities with sterile instruments. The skin was closed by means of two sutures with resorbable thread. Three mice per patient were implanted. When tumour growth reached 1 cm^3^ (within 4–6 months; the take rate was 30%), the mice were sacrificed, the tumours excised and viable tissue was cut into pieces and frozen as described. At the next step, pieces were thawed and implanted onto 16 mice per patient material. When the tumour reached about 10–20 mm^3^ in volume, the animals were randomized and treated as described above. Animal experiments were performed according to European Community directives for animal care (No. 86/609/EEC, November 24, 1986).

**The paper explained**

PROBLEM:

Conventional treatments often fail to provide long term survival in patient with pancreatic carcinoma and advanced cervical carcinoma, calling for new therapeutic options. Oncolytic virotherapy represents an exciting new biological approach to cancer treatment. The rat parvovirus H-1PV is one of the oncolytic viruses currently tested in clinical trials. As observed with other anticancer treatments, some tumour cells may be resistance to the intrinsic cytotoxicity of the oncolytic virus and be responsible for tumour relapse. In order to maximize the clinical benefit of H-1PV-based therapies, it is therefore especially important to devise combination strategies that synergistically enhance the oncosuppressive capacity of the virus while preserving its excellent safety profile.

RESULTS:

In this study, we show that sub-lethal doses of the histone deacetylase inhibitor valproic acid (VPA) enhance the oncolytic activity of H-1PV in a synergistic manner against a range of human cancer cell lines derived from patients with cervical and pancreatic carcinomas. This increase in tumour cell-killing is associated with accumulation of reactive oxygen species (ROS) and DNA damage, two important mediators of virus and VPA oncotoxicity, leading to increased apoptosis. At the molecular level we show for the first time that the parvovirus non-structural NS1 protein, the major effector of virus replication and cytotoxicity, is acetylated at two specific amino-acid residues (K85 and K257). NS1-dependent viral gene expression, virus multiplication and cell killing, are all enhanced upon VPA treatment, correlating with an increase in NS1-acetylation. Conversely, these NS1 activities are severely impaired by amino-acid substitutions of above NS1 acetylation sites. The synergistic effect of VPA on H-1PV oncolytic activity has been validated *in vivo* using three rodent xenograft tumour models. The combination regime leads to permanent eradication of established tumours in all co-treated animals with no apparent deleterious side-effects.

IMPACT:

H-1PV and VPA are both currently tested in clinical trials as monotherapy against various malignancies. The present study provides proof-of-concept for the design of an improved anticancer combination protocol to be tested in clinical studies against cervical and pancreatic carcinomas.

### Haematoxylin–eosin staining

Four-micrometer-thick paraffin sections were prepared from resected tumours and mounted on glass slides. For morphological examination, the H&E staining was carried out according to routine histological practice.

### Immunohistochemistry

Slides were analysed using a Ventana automat (Benchmark XT, Roche-Ventana, Tucson) according to the manufacturer's instructions. Immunostaining was performed on 4-µm-thick paraffin sections with the following specific antibodies: monoclonal anti-cleaved caspase-3 (Asp 175) (5A1E; Cell Signaling; used at 1:400 dilution), monoclonal anti-H2A.X Phospho-Histone (Ser 139) (clone 20E3, Cell Signaling, 1:100), anti-NS1 (1:500) and anti-VPs viral capsid protein (1:500). Immunohistochemical revelation was performed with the ultraView Universal DAB detection kit (Roche-Ventana). Negative controls were obtained by omitting the primary antibody. Slides were examined and pictures were taken with a Zeiss Axioskop 40 photomicroscope.

### *In situ* hybridization

A Ventana automat (Benchmark XT) was used according to the manufacturer's recommendations (XT INFORM HPV III iVIEW Blue). Briefly, after 8 min of proteinase III pre-treatment, paraffin sections were incubated with cocktails of biotinylated DNA probe directed against low-risk HPV (types 6 and 11, used as negative control) or high-risk HPV (types 16, 18, 31, 33, 35, 39, 45, 51, 52, 56, 58 and 66). An ISH iVEW Blue+ (Ventana) kit was used for detection.

### Immunofluorescence

Tissue samples were formalin-fixed for 72 h and embedded in paraffin blocks before being cut into 20-µm sections. Deparaffinized sections were treated with antigen retrieval solution (0.01 M sodium citrate buffer, pH 6.0) by boiling in a microwave oven for 10 min. The sections were then permeabilized with 0.1% Triton X, washed with PBS and blocked with 10% goat or donkey serum before incubation with primary antibodies at 4°C overnight. The primary antibodies used were mouse anti-γ-H2AX (1:200; Ab22551, Abcam), rabbit anti-NS1 SP8 antibody (1:500; Bodendorf et al, 1999), mouse anti-8-OH-dG (1:200; QED Bioscience, Inc., San Diego, CA). Alexa Fluor® 488 goat anti-mouse or Alexa Fluor® 594 donkey anti-rabbit secondary antibodies (1:500) (Invitrogen) were applied for 4 h at 4°C. Nuclei were counterstained with 1.5 µg/ml 4′,6-diamidino-2-phenylindole (DAPI) in Vectashield mounting medium (Vector Laboratories, Burlingame, CA). Images were acquired on a digital microscope (KEYENCE BZ-9000E, Japan) and analysed with BZ-II Analyzer software.

### Analysis of virus bio-distribution

RNA was prepared from cryosections (0.8 mm thick) of frozen organs (kidney, large intestine, small intestine, liver, brain, lung, heart) or tumours with TRIzol® Reagent RNA-extraction kit (Invitrogen) according to the manufacturer's instructions. After DNase digestion, 1 µg of total RNA was subjected to quantitative RT-PCR analysis as described above.

### Statistical analyses

#### Cell culture studies

LDH measurements from different cell lines were assumed to be independent. Therefore, statistical analysis was performed separately for each cell line. For each condition, arithmetic mean and standard deviation of cell lysis values were calculated and displayed graphically.

The following evaluations were carried out only for cervical and pancreatic derived cancer lines: Two-way analysis of variance (ANOVA) with interaction was conducted on a significance level of 5% to model cell lysis in dependence of H-1PV concentration (factor 1), treatment (VPA *vs*. no VPA, factor 2) and the interaction of concentration and treatment. *p*-values less than 0.05 in the *F*-test for the concentration x treatment interaction term were considered to demonstrate statistically significant synergistic behaviour of H-1PV and VPA in terms of cytotoxicity. The normality assumption of the ANOVA model was checked by exploring quantile–quantile (QQ) plots of the residuals of the model. If statistically significant synergism of H-1PV and VPA could be concluded, two-sided two sample Welch t-tests were performed to compare, for each H-1PV concentration, conditions with and without VPA. *p*-values obtained from two-sided two sample Welch t-tests were adjusted for multiple testing using the Bonferroni method. Adjusted *p*-values less than 0.05 were regarded as statistically significant.

#### Animal studies

Statistical analysis was performed separately for HeLa and AsPC-1 xenografts. For each treatment group and each observation time point, the arithmetic mean and the corresponding standard deviation of the tumour volumes of all rats (*n* = 8) belonging to each group was calculated. Kaplan–Meier survival analysis was applied to compute survival curves for selected treatment groups considering as death point the first time point (in days) at which the tumour reached a volume of ≥4000 mm^3^ (=day at which animals were sacrificed). Rats for which the tumour volume did not reach 4000 mm^3^ within the observation period of maximum 80 days were considered censored observations. Obtained Kaplan–Meier survival curves were slightly modified to yield a more informative graphical representation. Finally, the two-sided log-rank (Mantel-Cox) test was conducted on a global significance level of 5% to compare Kaplan–Meier survival curves between selected pairs of treatment groups. Obtained *p*-values were adjusted for multiple testing using the Bonferroni method. Adjusted *p*-values less than 0.05 were regarded as statistically significant. Statistical analysis was carried out with the open-source statistical software environment R, version 2.14.1 (http://www.R-project.org).

## Supplementary material

As a service to our authors and readers, this journal provides supporting information supplied by the authors. Such materials are peer reviewed and may be re-organized for online delivery, but are not copy-edited or typeset. Technical support issues arising from supporting information (other than missing files) should be addressed to the authors.
