# Supplementary material for: Synergistic combination of valproic acid and oncolytic parvovirus H-1PV as a potential therapy against cervical and pancreatic carcinomas
Source: EMBO Mol Med. 2013 Sep 17;5(10):1537–55. doi: 10.1002/emmm.201302796 (PMC3799578; doi:10.1002/emmm.201302796)
Supplement: Supplementary file 1 [file emmm0005-1537-SD1.pdf]

# SUPPORTING INFORMATION

**Synergistic combination of valproic acid and oncolytic parvovirus H-1PV as a potential therapy against cervical and pancreatic carcinomas *(Li et al.)***

## Table of Contents


**Supporting Information Table S1.** Summary of statistical analysis results obtained for cancer cell lines co-treated with H-1PV and VPA……………………………………………………3

**Supporting Information Table S2.** Summary of statistical analysis results obtained for HeLa and AsPC-1 animal experiments……………………………………………………………...4

**Supporting Information Figure S1.** VPA stimulates H-1PV-mediated oncolysis in cervical and pancreatic derived cancer cell lines…………………..…………………………………………………………………5

**Supporting Information Figure S2.** H-1PV/VPA co-treatment is not harmful to normal primary human cells……………………………………………………………………………...6

**Supporting Information Figure S3.** The HDAC inhibitor NaB synergizes with H-1PV in killing HeLa cells by inducing ROS accumulation and DNA damage……………………………………7

**Supporting Information Figure S4.** NaB increases NS1-mediated cytotoxicity………………...8

**Supporting Information Figure S5.** NaB treatment increases H-1PV expression...………………………………………………………………………………...9

**Supporting Information Figure S6.** VPA increases H-1PV production in cervical carcinoma derived cell lines…………………………………………………………………….....10

**Supporting Information Figure S7.** VPA enhances H-1PV oncosuppressive capacity in the HeLa xenograft rat model…………………………………………………………..…11-12

**Supporting Information Figure S8.** Virus biodistribution in H-1PV/VPA-co-treated animals after tumour eradication……………………………………………………………………..13

**Supporting Information Figure S9.** Analysis of HeLa all-derived tumours for the presence of the human papillomavirus genome……………………………………......……………………………………….14

**Supporting Information Figure S10.** Complete remission of established AsPC-1 tumours upon combined treatment with H-1PV and VPA……………………………………………………….15

**Supporting Infomation Figure S11.** Morphological analysis of AsPC-1 cell tumour xenografts…………………………………………………………………………………....16

**Supporting Information Figure S12.** H-1PV/VPA co-treatment leads to complete eradication of established primary tumour material xenografts……………………………………………...17-18

**Supporting Information Table S1. Summary of statistical analysis results obtained for cancer cell lines co-treated with H-1PV and VPA.**

| **VPA 1 mM** | |
|---|---|
| Cell line | P-value* |
| HeLa | 1.491E-10 |
| CaSki | 8.590E-07 |
| SiHa | 3.488E-07 |
| CxCa | 1.617E-04 |
| T3M-4 | 5.44E-05 |
| MiaPaCa-2 | 6.134E-07 |
| AsPC-1 | 2.262E-07 |
| **VPA 0.5 mM** | |
| Cell line | P-value* |
| HeLa | 2.90E-09 |
| CaSki | 1.79E-06 |
| SiHa | 2.15E-10 |
| CxCa | 4.376E-03 |
| T3M-4 | 5,47E-08 |
| MiaPaCa-2 | 6.405E-04 |
| AsPC-1 | 6.63E-06 |

*Calculated from two-way ANOVA with interaction. P-values were computed from F-tests conducted to evaluate, separately for each cell line, the size of the interaction effect of the tested H-1PV and VPA concentrations. All P-values are smaller than 0.05, indicating statistically significant synergistic cell killing of H-1PV and VPA combinations.

**Supporting Information Table S2. Summary of statistical analysis results obtained for HeLa and AsPC-1 animal experiments.**

| **HeLa xenograft model** | | |
|---|---|---|
| Group 1 | Group 2 | P-value* |
| control | VPA | 1.0000 |
| control | H-1PV 2.5 x 10^8 | 0.4866 |
| control | H-1PV 1.25 x 10^8 + VPA | **0.0006** |
| VPA | H-1PV 2.5 x 10^8 | 1.0000 |
| VPA | H-1PV 1.25 x 10^8 + VPA | **0.0003** |
| H-1PV 2.5 x 10^8 | H-1PV 1.25 x 10^8 + VPA | **0.0002** |
| **AsPC-1 xenograft model** | | |
| Group 1 | Group 2 | P-value* |
| control | VPA | 0.8520 |
| control | H-1PV 2.5 x 10^8 | 0.1230 |
| control | H-1PV 2.5 x 10^8 + VPA | **0.0003** |
| VPA | H-1PV 2.5 x 10^8 | 1.0000 |
| VPA | H-1PV 2.5 x 10^8 + VPA | **0.0005** |
| H-1PV 2.5 x 10^8 | H-1PV 2.5 x 10^8 + VPA | **0.0032** |

***** P-values were calculated from two-sided log-rank tests and adjusted for multiple testing by application of the Bonferroni Method. Adjusted P-values in bold are regarded as statistically significant. N=8 rats were studied in each group.

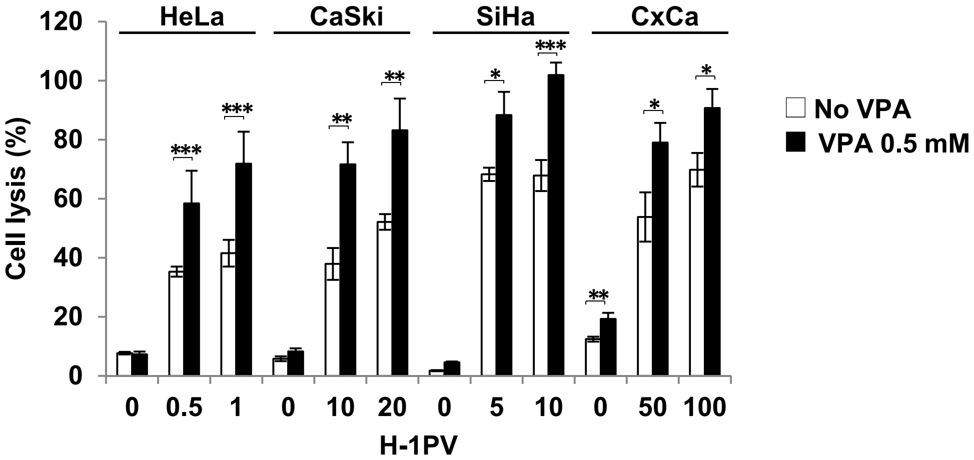


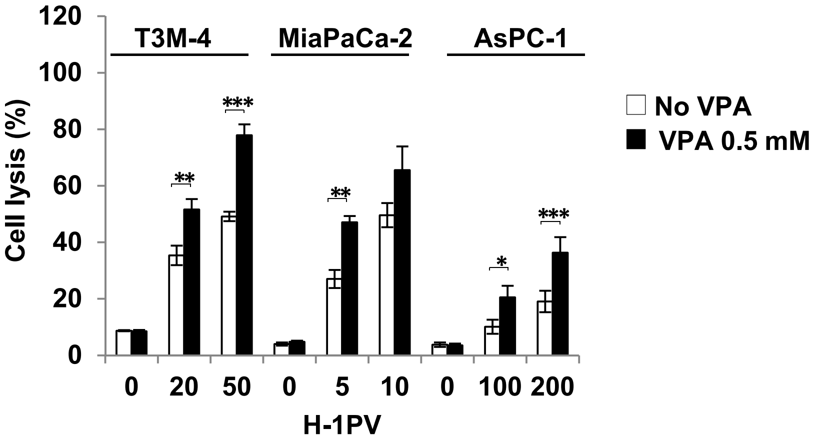


**Supporting Information Figure S1. VPA stimulates H-1PV-mediated oncolysis in cervical and pancreatic derived cancer cell lines.** Cancer cell lines were seeded into 96-well plates and infected with H-1PV at the indicated MOI (pfu/cell) in combination or not with VPA (0.5 mM). After 72 h of incubation, percentages of lysed cells were assessed by LDH assay, as described in Fig 1A. Columns show average cell lysis values with standard deviation bars. Results from a typical experiment performed in quadruplicate are shown.
*P < 0.05; **P < 0.01; ***P < 0.001 as calculated by two sample Welch t-test and adjustment for multiple tests with the Bonferroni method.

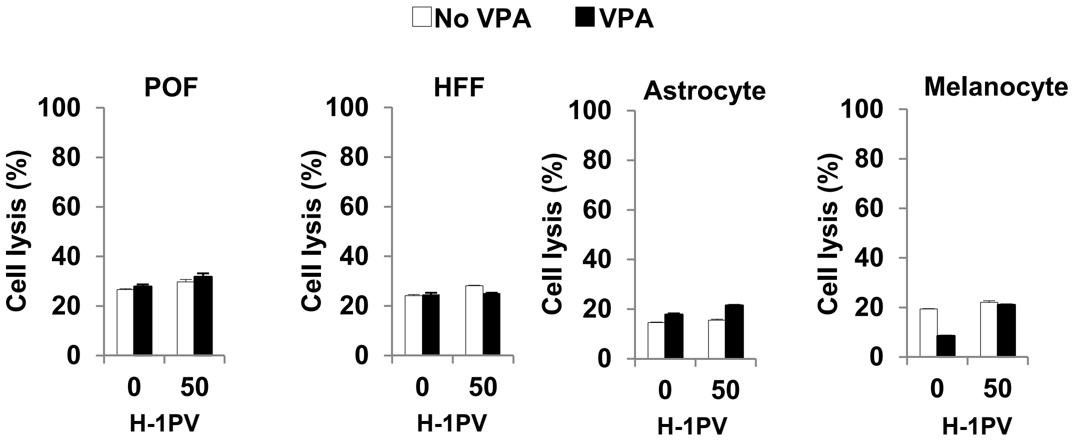


**Supporting Information Figure S2. H-1PV/VPA co-treatment is not harmful to normal primary human cells.** Human primary oral fibroblasts (POF), human foreskin fibroblasts (HFF), human astrocytes, and human melanocytes were seeded into 96-well plates and either mock-treated or infected with H-1PV (MOI of 50 pfu/cell) in the presence (black bars) or absence (white bars) of VPA (1 mM). Cell lysis was measured by LDH assay 72 h post-treatment as described in Fig 1A. Columns show average cell lysis values with standard deviation bars, calculated from four independent replicates per experimental condition.

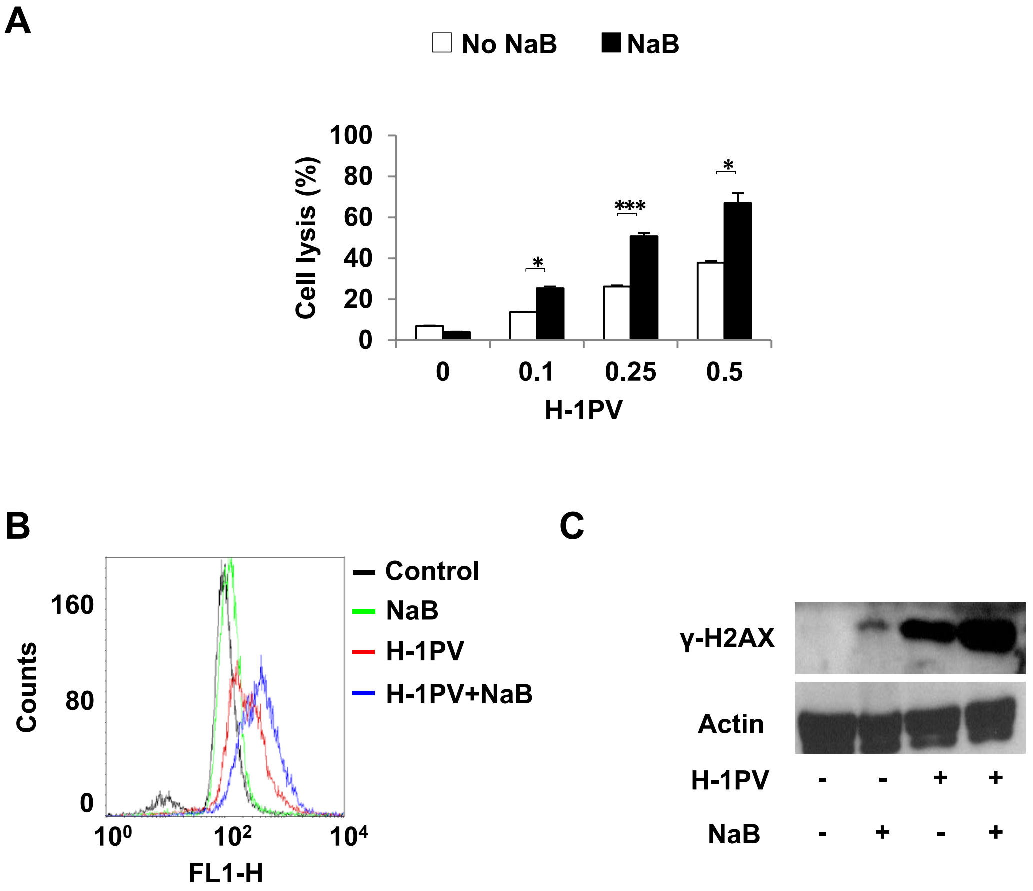


**Supporting Information Figure S3. The HDAC inhibitor NaB synergizes with H-1PV in killing HeLa cells by inducing ROS accumulation and DNA damage. (A)** LDH assay. HeLa cells were seeded into 96-well plates and infected with H-1PV at the indicated MOI (pfu/cell) in combination or not with NaB (1 mM). After 72 h of incubation, percentages of lysed cells were assessed by LDH assay, as described in Fig 1A. Columns show average cell lysis values with standard deviation bars of a typical experiment performed in quadruplicate. *$P < 0.05$; **$P < 0.01$; ***$P < 0.001$ as calculated by two sample Welch t-test and adjustment for multiple tests with the Bonferroni method. **(B)** ROS content. Representative FACS-plots of DCFH-DA-stained HeLa cells, untreated (black) or treated with NaB alone (1 mM, green), with H-1PV alone (MOI of 1 pfu/cell, red) or with both H-1PV and NaB (blue). FACS analysis was performed 24 h post-treatment as described in Fig 1B. A minimum of 20,000 events was acquired. **(C)** DNA damage. Western blotting analysis of the protein levels of phosphorylated H2AX (γ-H2AX) in HeLa cells left untreated (-) or treated with NaB (1 mM), H-1PV (1 pfu/cell), or both. 20 µg of total cell lysate were used for analysis. Actin was used as a loading control.

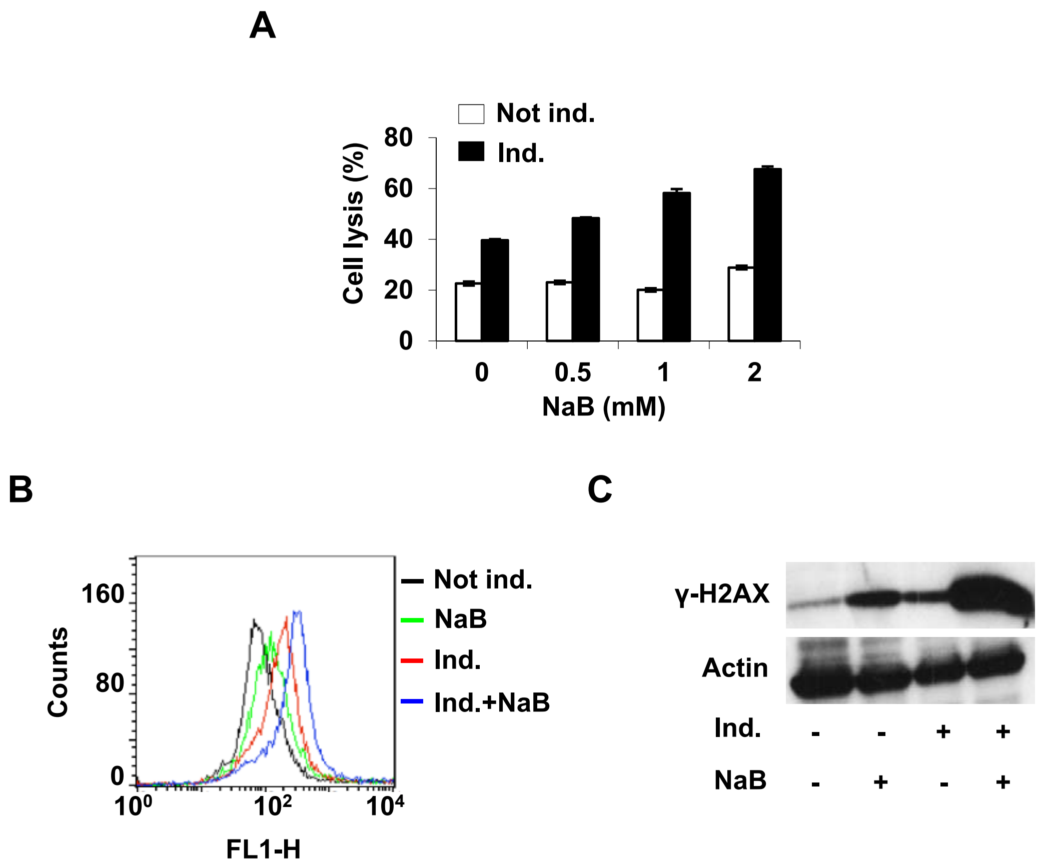


**Supporting Information Figure S4. NaB increases NS1-mediated cytotoxicity. (A)** LDH assay. HeLa-NS1 stably transfected cells were grown with (Ind.) or without (Not ind.) doxycycline (1 µg/ml) and with NaB at the indicated concentration (mM) for 72 h before being processed for LDH assay. **(B)** ROS content. HeLa-NS1 cells were grown in medium supplemented (Ind.) or not (Not ind.) with doxycycline and treated with NaB (0.5 mM) for 24 h before being analysed for ROS content by flow cytometry. **(C)** DNA damage. Lysates from mock-treated or doxycycline-induced cells incubated with or without NaB (1 mM) were subjected to Western blotting analysis using antibodies specific for γ-H2AX and actin (loading control).

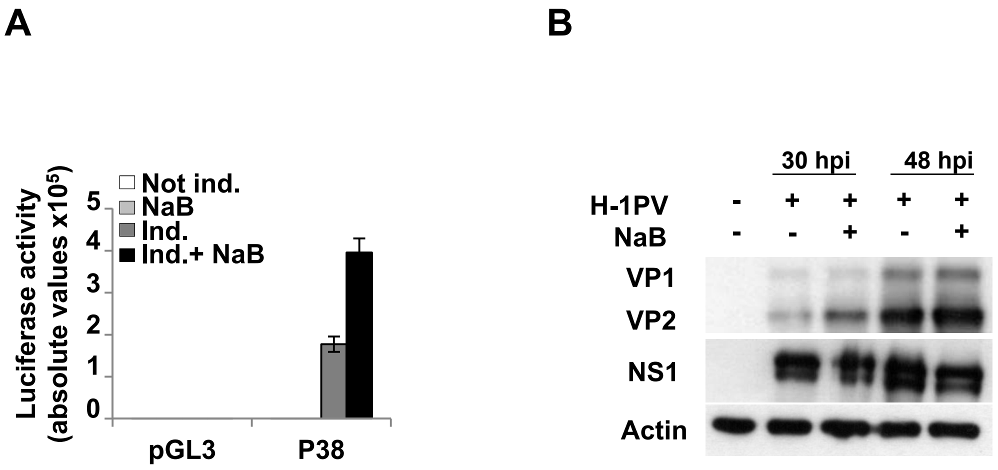


**Supporting Information Figure S5. NaB treatment increases H-1PV expression. (A)** NaB treatment enhances NS1 transcriptional activities. HeLa-NS1 cells were transfected with the pGL3-H-1PV-P38 and pGL3 basic plasmids. Transfected cells were grown in medium containing or not containing doxycycline (1 µg/ml) in presence or absence of NaB (1 mM). 48 h later, the cells were lysed and subjected to Dual Luciferase Assay as described in Fig 3B. Values represent the mean ratios of *firefly* activity to protein concentration with standard deviation bars, from three replicates. **(B)** NaB treatment increases viral protein synthesis. HeLa cells were infected with H-1PV (MOI of 1 pfu/cell) and grown for 30 and 48 h in the presence or absence of NaB (1 mM). Cell lysates were analysed by Western blotting for the presence of viral NS1 and capsid proteins (VP1 and VP2). Actin was used as a loading control.

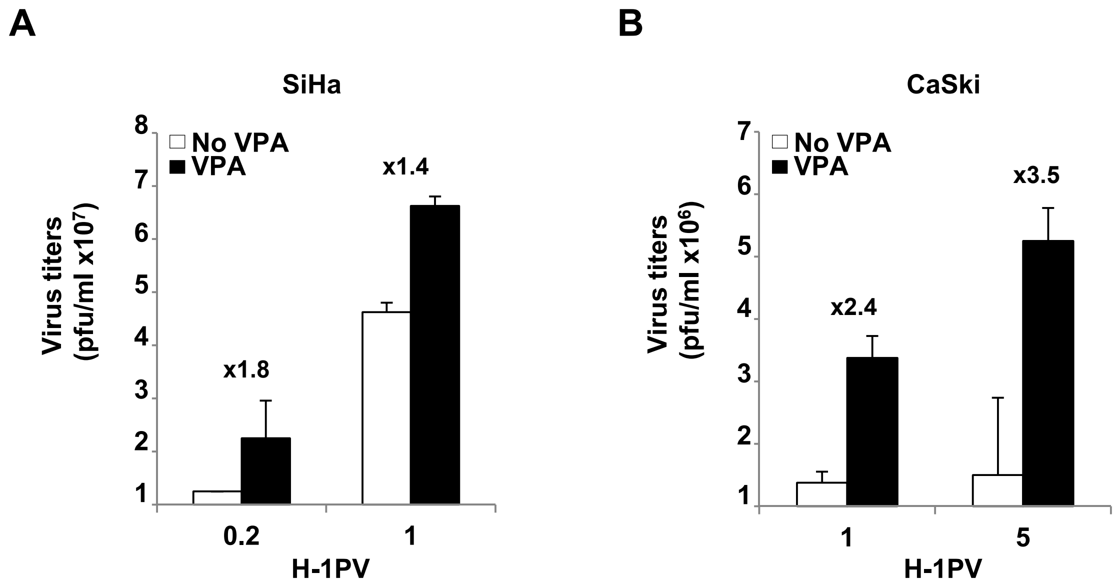


**Supporting Information Figure S6. VPA increases H-1PV production in cervical carcinoma derived cell lines. (A)** SiHa and **(B)** CaSki cells were infected with H-1PV at the indicated MOIs (pfu/cell), either alone (white bars) or in combination with VPA (1 mM, black bars). At 96 h, cells were harvested and total cell lysates were analyzed by plaque assay to determine the yields of infectious viruses. Numbers on top of the columns indicate the fold increase in virus titers in presence versus absence of VPA.

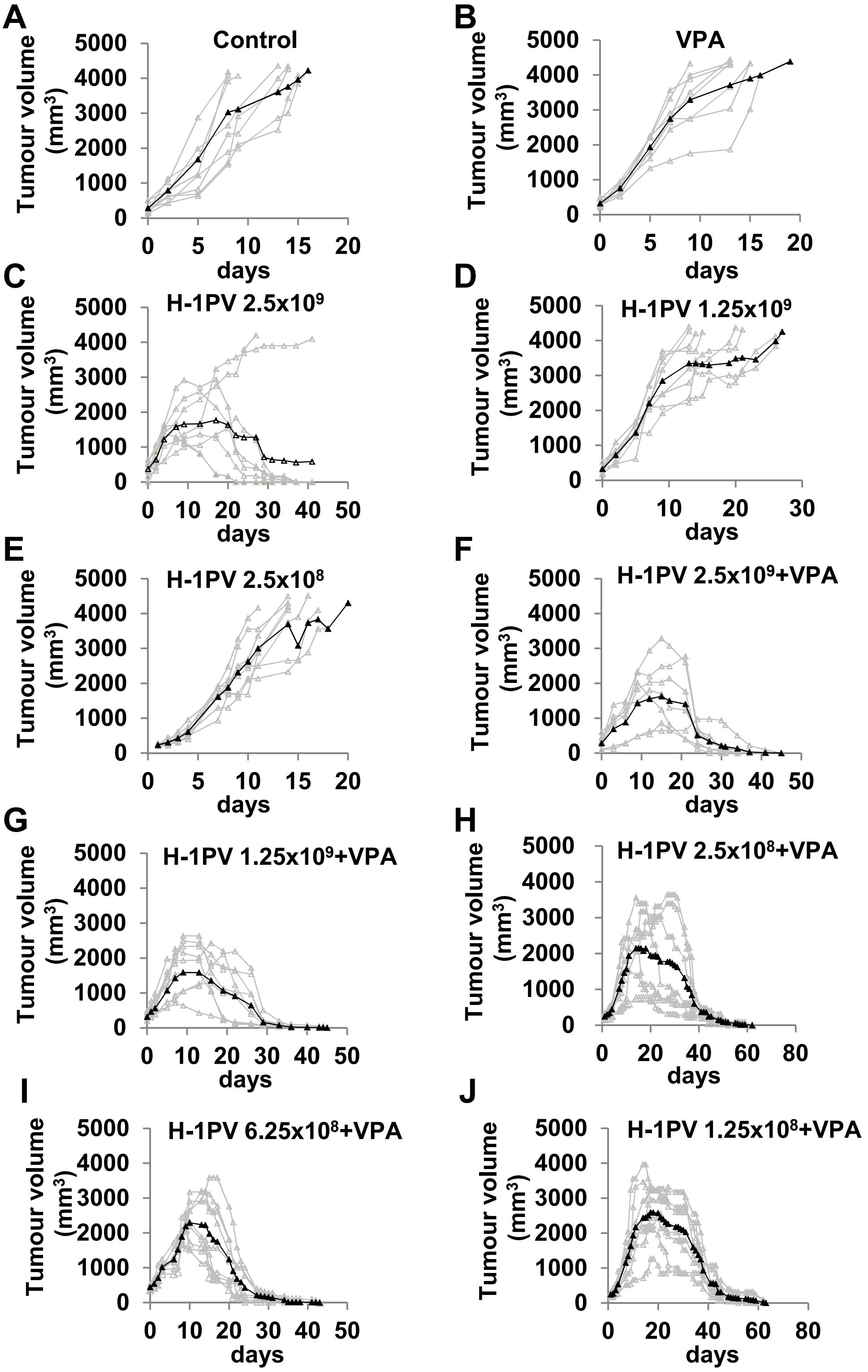
A
Control
Tumour volume (mm3)
days
B
VPA
C
H-1PV 2.5x10^9
D
H-1PV 1.25x10^9
E
H-1PV 2.5x10^8
F
H-1PV 2.5x10^9+VPA
G
H-1PV 1.25x10^9+VPA
H
H-1PV 2.5x10^8+VPA
I
H-1PV 6.25x10^8+VPA
J
H-1PV 1.25x10^8+VPA

**Supporting Information Figure S7. VPA enhances H-1PV oncosuppressive capacity in the HeLa xenograft rat model. (A-J)** Growth curves of HeLa cell tumour xenografts. Nude rats were treated as described in Fig 8A. H-1PV was used at the indicated doses (pfu/cell) and VPA at 100 mg/kg. Animals were sacrificed when the tumour volume reached 4000 $mm^3$. Each grey line shows the evolution of tumour volume in a single rat during the time of investigation. Black lines indicate mean tumour volume from all animals in their respective experimental groups.

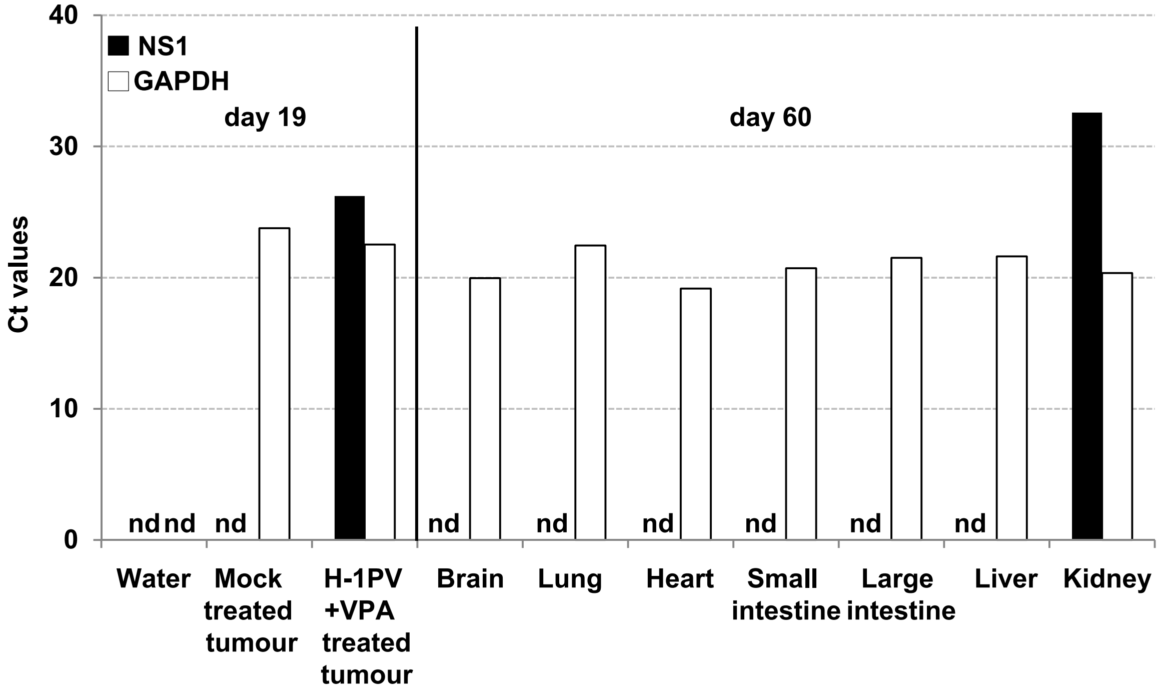


**Supporting Information Figure S8. Virus biodistribution in H-1PV/VPA-co-treated animals after tumour eradication.** HeLa tumour-bearing rats from the mock-treated (control) and H-1PV/VPA co-treated groups were sacrificed on day 19. A second co-treated animal was sacrificed on day 60, while being tumour-free for 20 days, to determine possible residual expression of the viral NS1 gene in the indicated organs. Total RNA was extracted from cryosections of frozen organs (0.8 mm thick) and reverse transcribed. cDNAs were used as templates for quantitative PCR with primer pairs specific for GAPDH (white bars, used for normalization) and NS1 (black bars). PCR results are shown as Ct-values. Nd, below the detection limit.

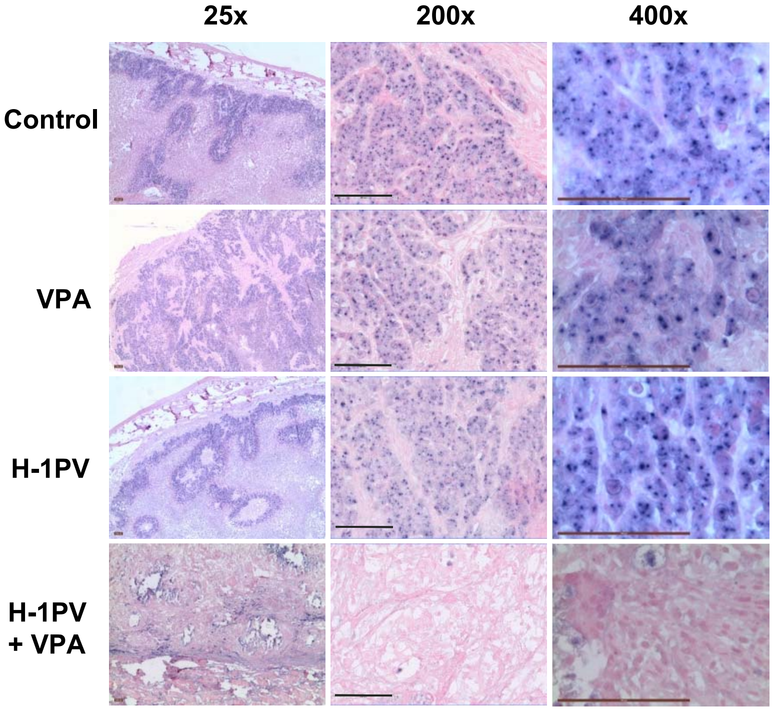


**Supporting Information Figure S9. Analysis of HeLa all-derived tumours for the presence of the human papillomavirus genome.** Tumours from rats treated or not with H-1PV and/or VPA were resected, fixed in 4% buffered formalin, and paraffin embedded. 20 µm-thick sections were used for *in situ* hybridization analysis of HPV 18 DNA, which is integrated into the genome of HeLa cells. High-risk HPV 18 DNA was detected in the nuclei of cells forming the tumour mass of control, VPA-treated and H-1PV-treated animals. Only a few residual positive cells were detected in H-1PV/VPA co-treated tumours after 19 days of treatment.

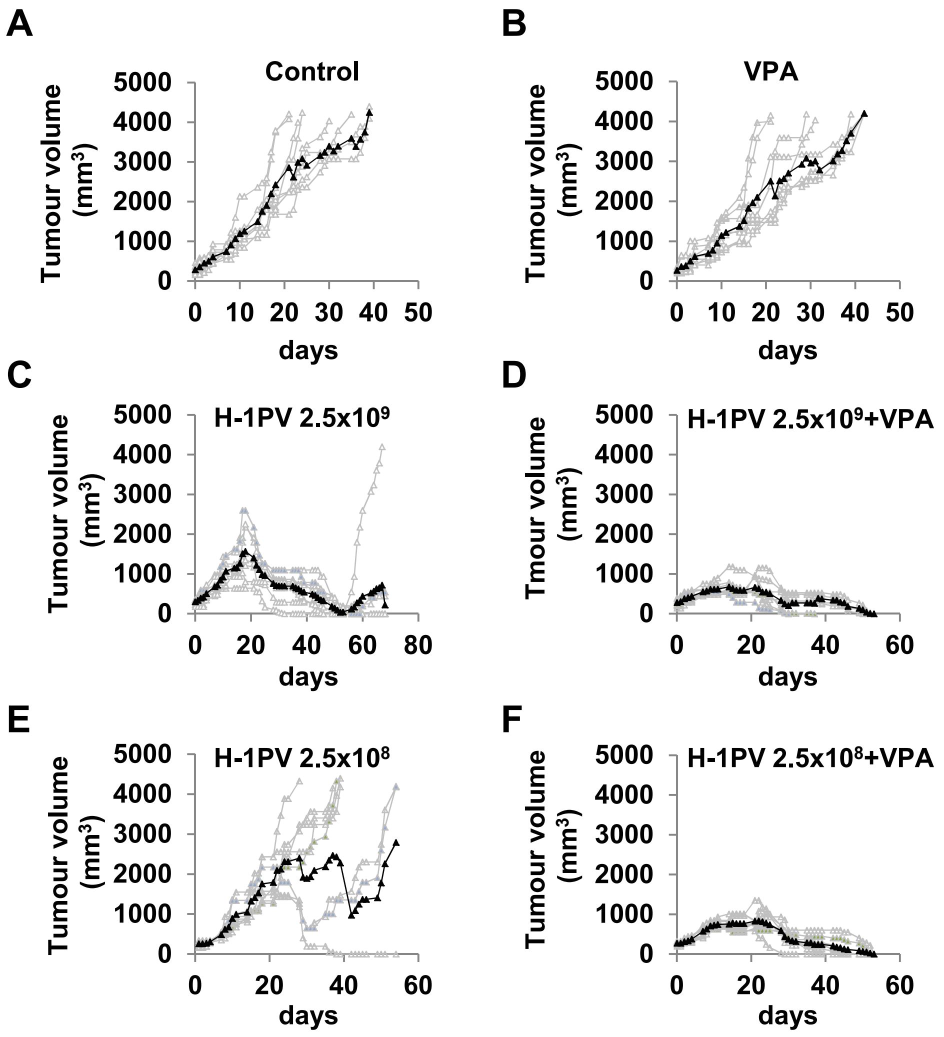


**Supporting Information Figure S10. Complete remission of established AsPC-1 tumours upon combined treatment with H-1PV and VPA. (A-F)** Growth curves of AsPC-1 cell-derived tumour xenografts. Nude rats were treated as described in Fig 9A. H-1PV was used at the indicated doses (pfu/cell), and VPA at 100 mg/kg. Animals were sacrificed when tumour diameter reached 4000 mm$^3$. Each grey line represents tumour development in a single rat, black line indicates mean tumour volume from all animals within the treatment group.

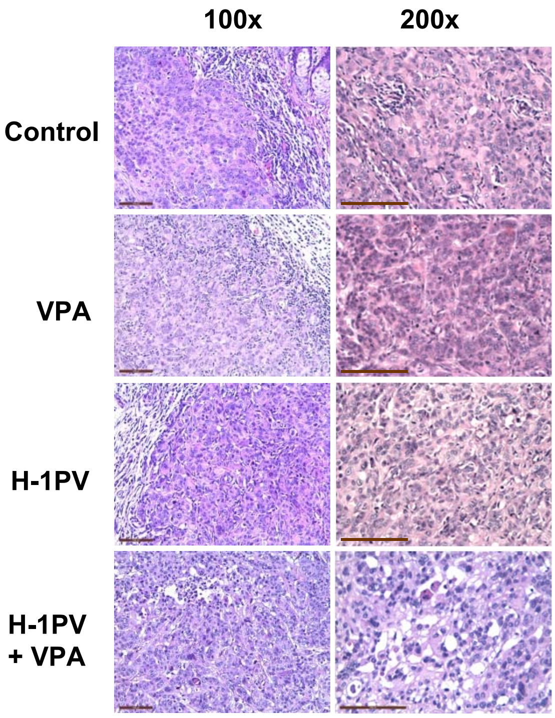


**Supporting Infomation Figure S11. Morphological analysis of AsPC-1 cell tumour xenografts.** Animals treated as in Fig 9A were sacrificed on day 27 and tumours were resected, fixed, and paraffin embedded. 4 µm sections were hematoxylin and eosin stained for histological analysis.

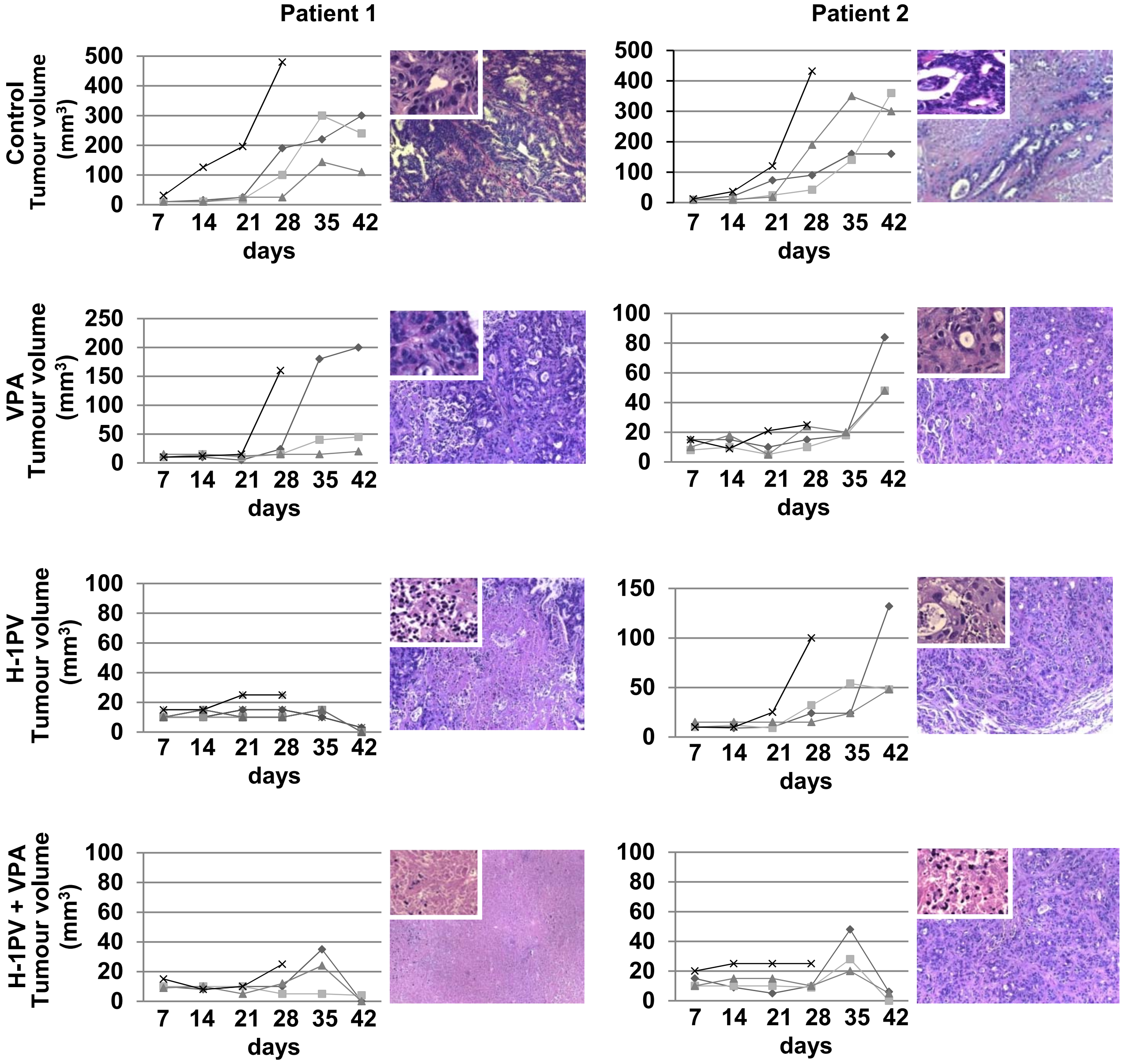

Patient 1
Patient 2
Control
Tumour volume
(mm³)
VPA
Tumour volume
(mm³)
H-1PV
Tumour volume
(mm³)
H-1PV + VPA
Tumour volume
(mm³)
days
7 14 21 28 35 42

**Supporting Information Figure S12. H-1PV/VPA co-treatment leads to complete eradication of established primary tumour material xenografts.** Six-week-old NOD/SCID mice (16 per patient x 2 patients) were implanted subcutaneously with patients' tumour material, expanded beforehand through one passage in the same animal strain. When the tumours reached 10-20 $mm^3$ in size (about 1 month after implantation), the mice were divided into four groups (4 animals/patient/group) and respectively mock-treated [saline injection 1x weekly intratumourously (i. t.) and 3x weekly intraperitoneally (i. p.)] or treated with VPA (100 mg/kg i.p. 3x weekly injections), H-1PV (i.t. injections of $1x10^9$ pfu/animal 1x weekly), or H-1PV/VPA combination. Treatment was continued for 30 days. Tumour size was measured weekly and animals were sacrificed when the tumour volume reached 500 $mm^3$. Each curve represents time-dependent tumour volume development in an individual animal. One animal from each group was sacrificed for analysis after 28 days. Tumour specimens were stained with hematoxylin and eosin at the end of the experiment. Representative micrographs (10x and 63x magnification) for each group are shown.
