# Supplementary figures and images for: Synergistic combination of valproic acid and oncolytic parvovirus H-1PV as a potential therapy against cervical and pancreatic carcinomas
Source: EMBO Mol Med. 2013 Sep 17;5(10):1537–55. doi: 10.1002/emmm.201302796 (PMC3799578; doi:10.1002/emmm.201302796)

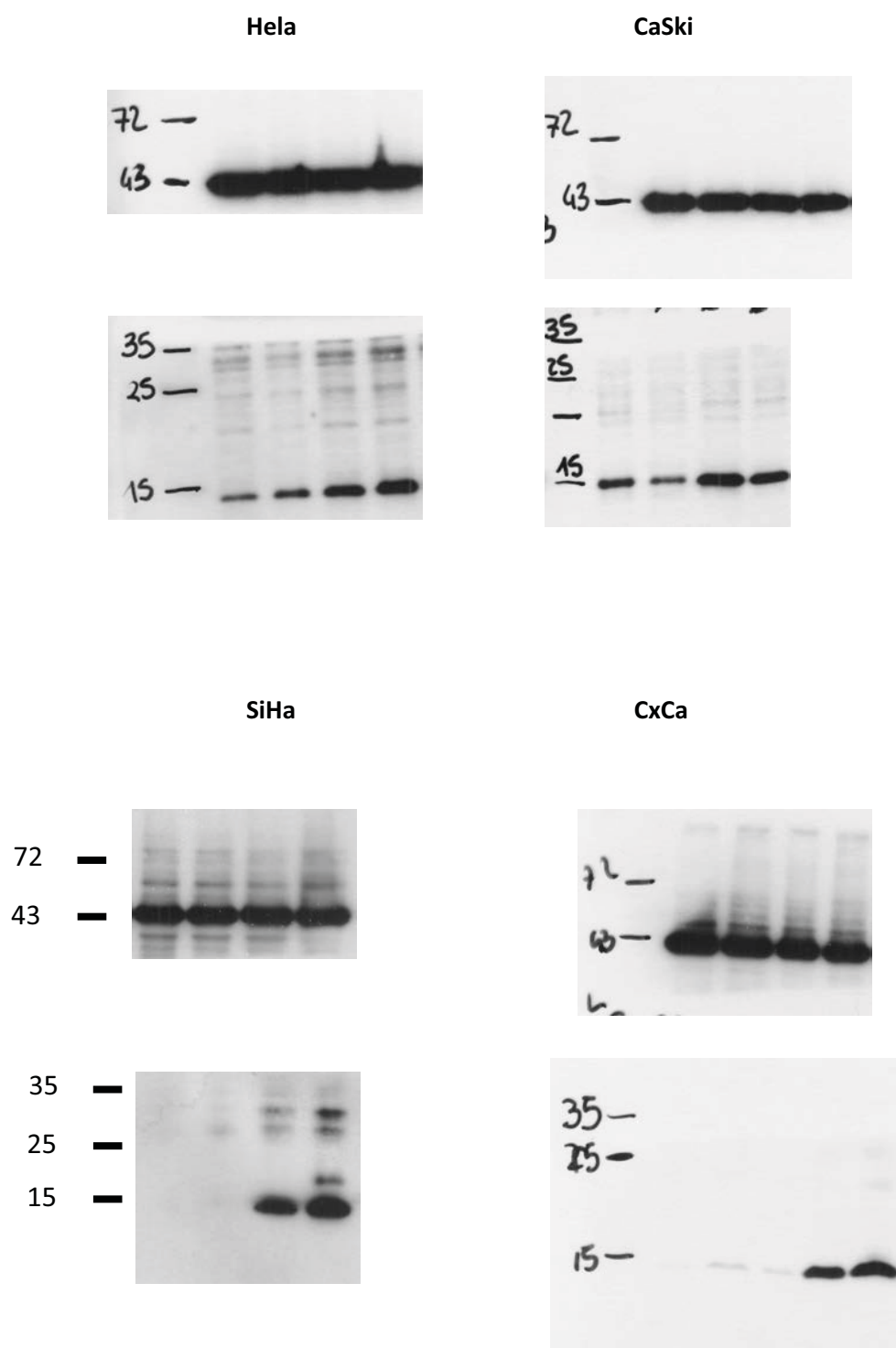

**Fig. 1C**

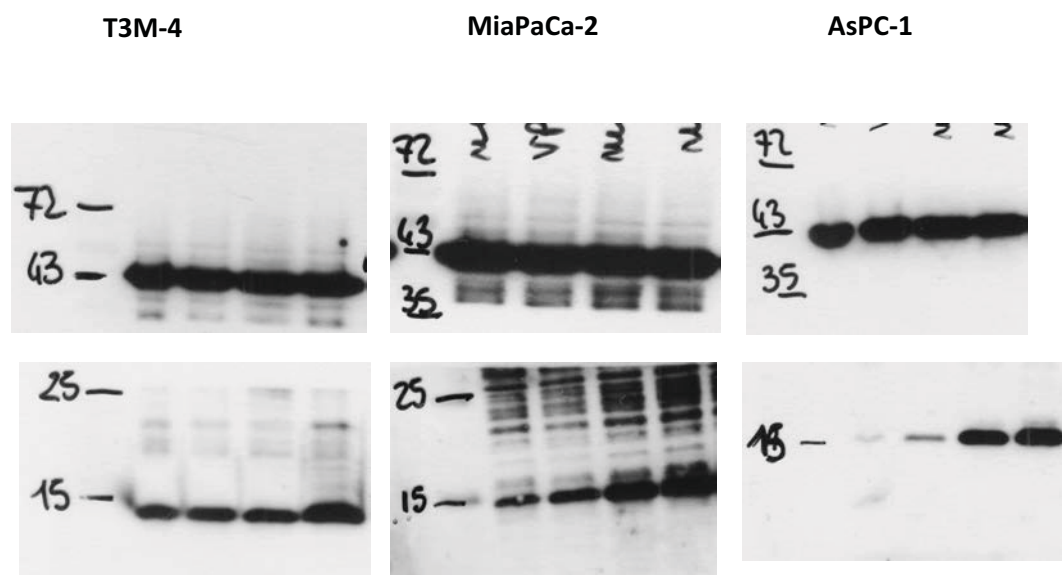

Fig. 1C

Supplement: Supplementary file 2 [file emmm0005-1537-SD2.pdf]

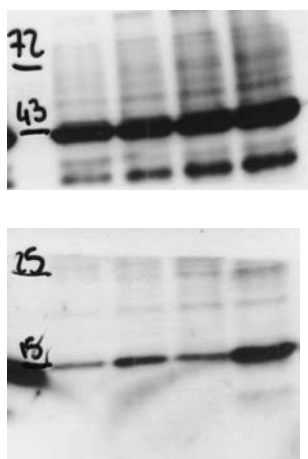

**Fig. 2C**

Supplement: Supplementary file 3 [file emmm0005-1537-SD3.pdf]

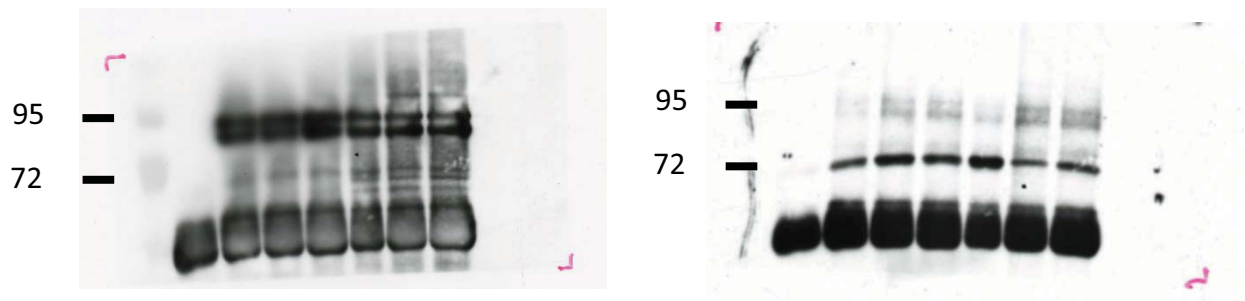

**Fig. 3A**

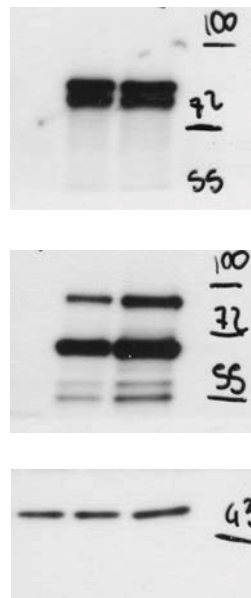

**Fig. 3D**

Supplement: Supplementary file 4 [file emmm0005-1537-SD4.pdf]

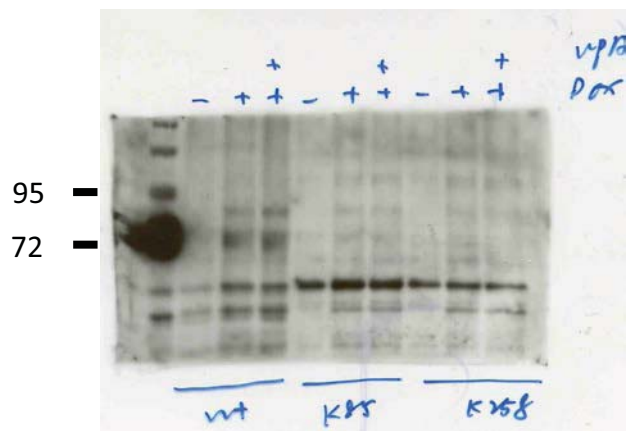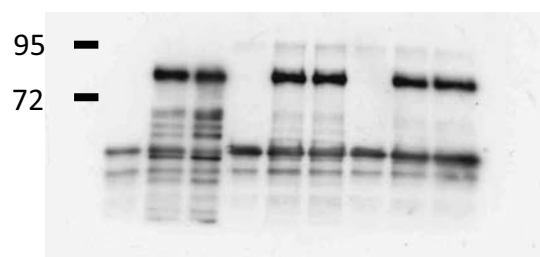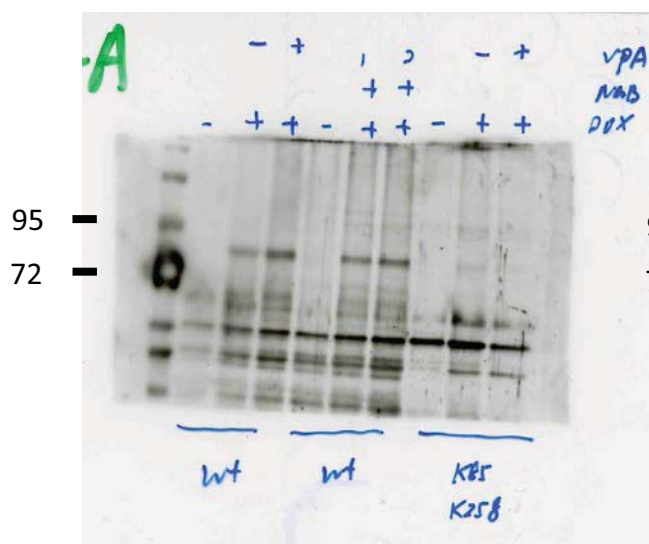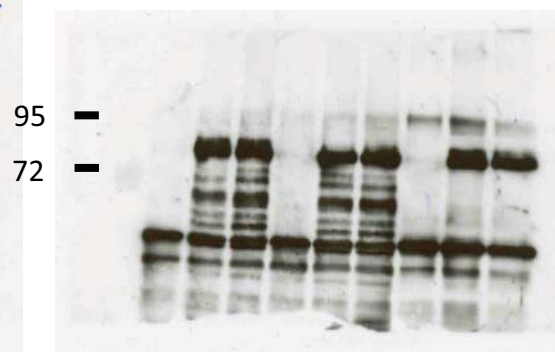

Fig. 5A and C

Supplement: Supplementary file 5 [file emmm0005-1537-SD5.pdf]

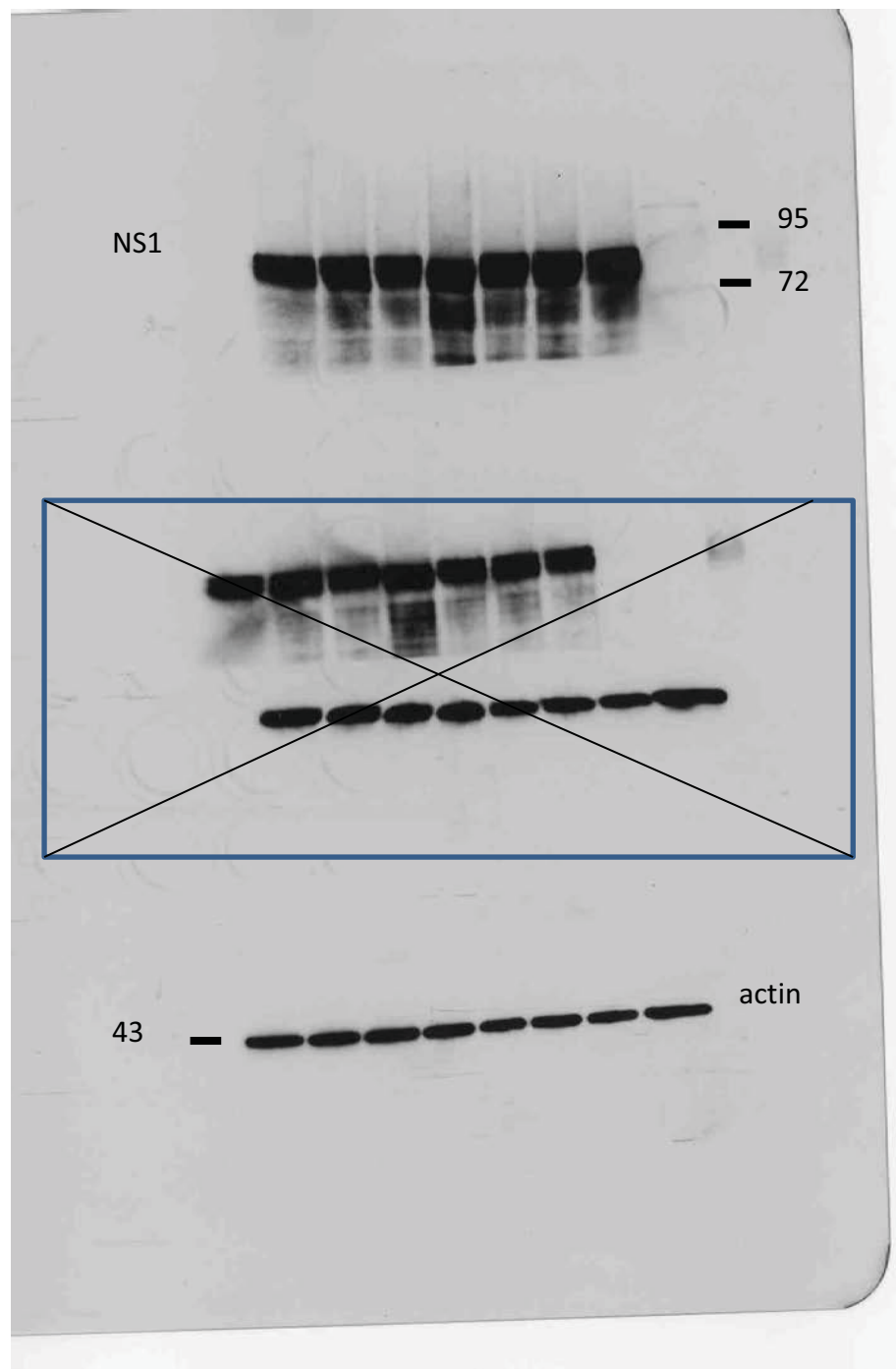

**Fig. 6A**

Supplement: Supplementary file 6 [file emmm0005-1537-SD6.pdf]

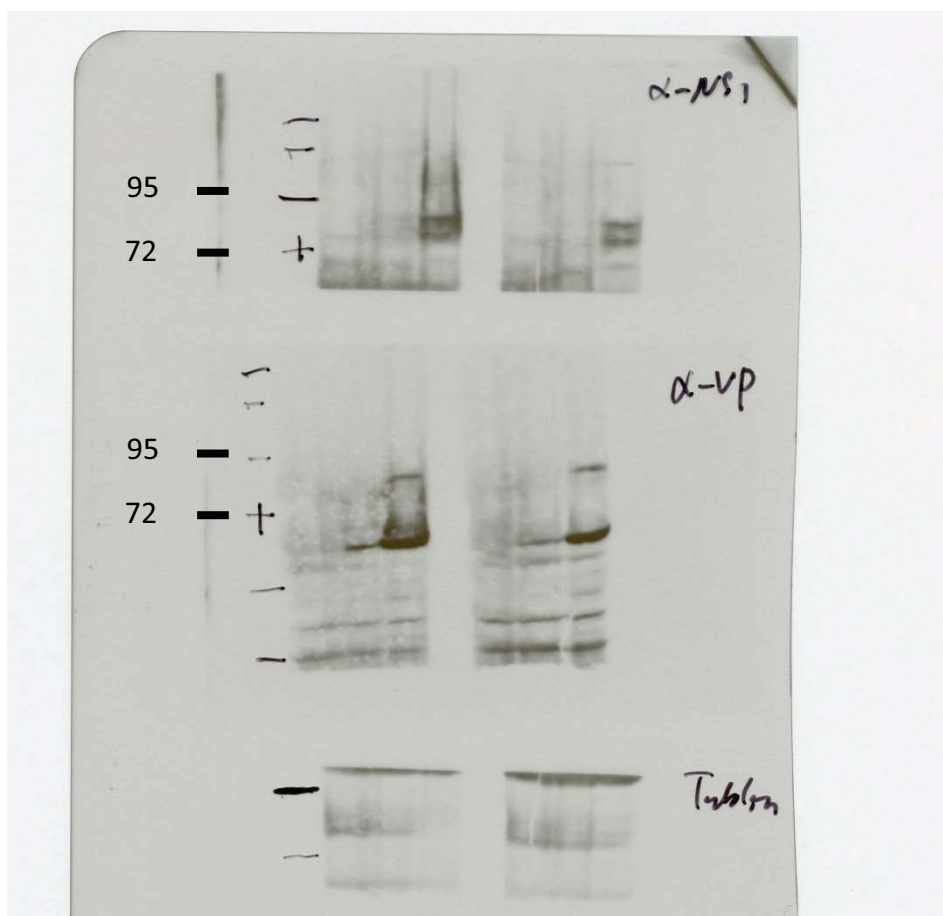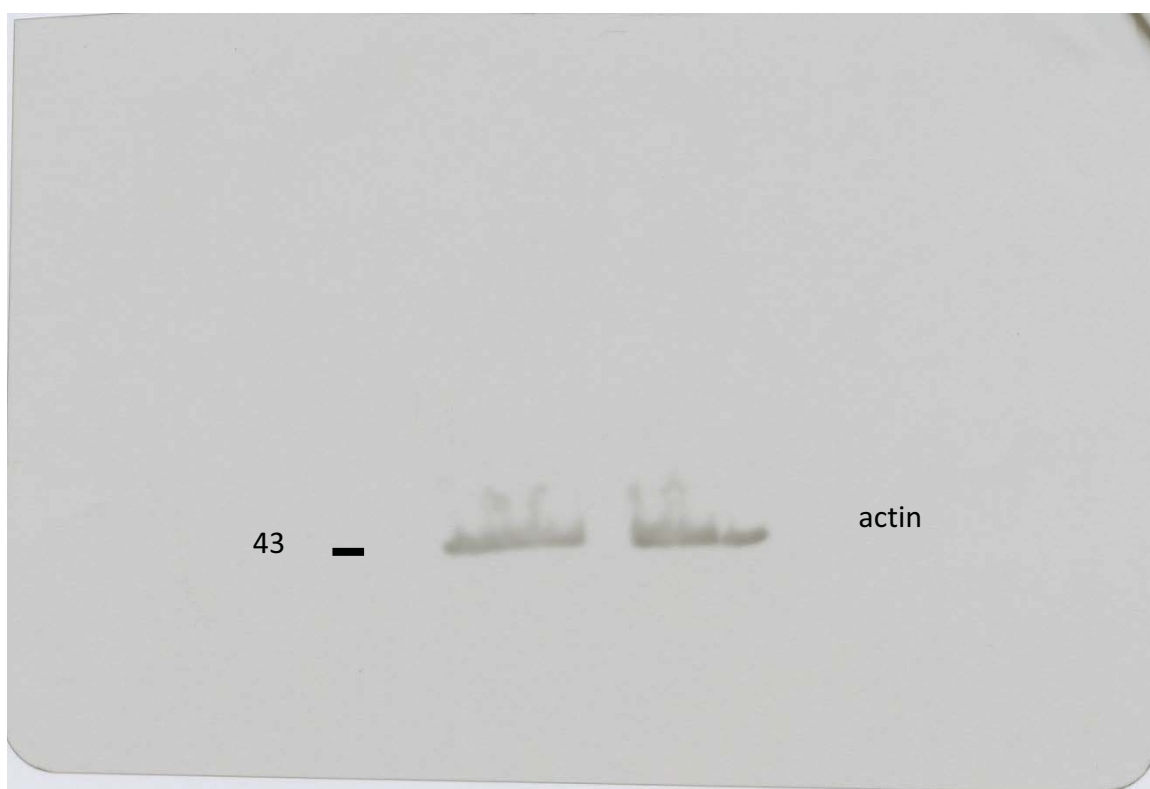

Fig. 8D and Fig. 9D

Supplement: Supplementary file 7 [file emmm0005-1537-SD7.pdf]

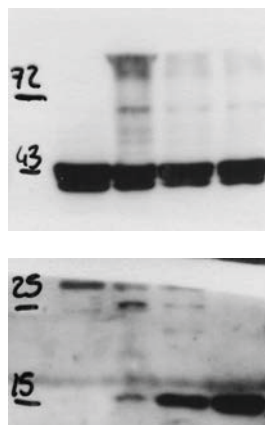

**Supporting information Fig. S3**

Supplement: Supplementary file 8 [file emmm0005-1537-SD8.pdf]

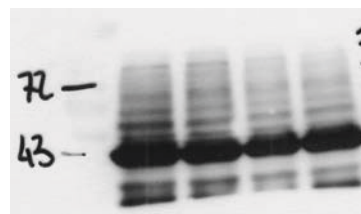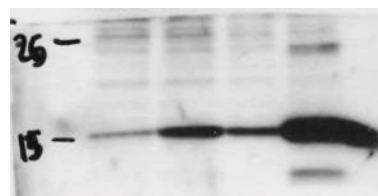

**Supporting information Fig. S4**

Supplement: Supplementary file 9 [file emmm0005-1537-SD9.pdf]

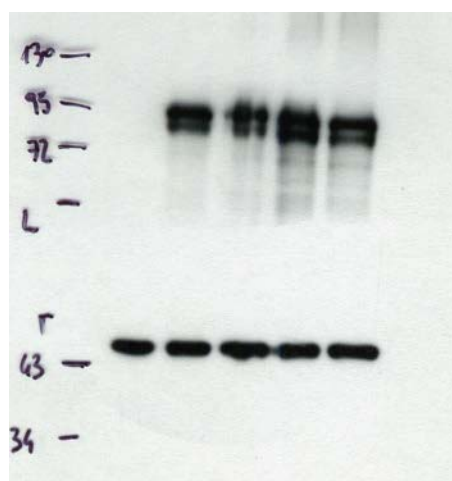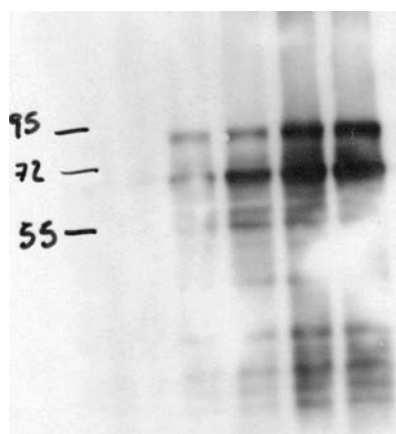

Supporting information Fig. S5

Supplement: Supplementary file 10 [file emmm0005-1537-SD10.pdf]
