# Supplementary material for: Synergistic combination of valproic acid and oncolytic parvovirus H-1PV as a potential therapy against cervical and pancreatic carcinomas
Source: EMBO Mol Med. 2013 Sep 17;5(10):1537–55. doi: 10.1002/emmm.201302796 (PMC3799578; doi:10.1002/emmm.201302796)
Supplement: Supplementary file 11 [file emmm0005-1537-SD11.pdf]

EMBO Molecular Medicine

Synergistic Combination of Valproic Acid and Oncolytic Parvovirus H-1PV as a Potential Therapy against Cervical and Pancreatic Carcinomas

Junwei Li, Serena Bonifati, Georgi Hristov, Tiina Marttila, Séverine Valmary-Degano, Sven Stanzel, Martina Schnˆlzer, Christiane Mougin, Marc Aprahamian, Svitlana P. Grekova, Zahari Raykov, Jean Rommelaere and Antonio Marchini

Corresponding author: Antonio Marchini, German Cancer Research Center DKFZ

Review timeline:

Submission date:	25 March 2013
Editorial Decision:	25 April 2013
Revision received:	23 July 2013
Editorial Decision:	05 August 2013
Revision received:	05 August 2013
Accepted:	07 August 2013

Transaction Report:

(Note: With the exception of the correction of typographical or spelling errors that could be a source of ambiguity, letters and reports are not edited. The original formatting of letters and referee reports may not be reflected in this compilation.)

Editor: Céline Carret

1st Editorial Decision 25 April 2013

Thank you for the submission of your manuscript to EMBO Molecular Medicine. We have now heard back from the three referees whom we asked to evaluate your manuscript. Although the referees find the study to be of potential interest, they also raise a number of concerns that need to be addressed in a major revision of your manuscript.

As you will see from the enclosed reports, the referees find the study interesting and comprehensive. While ref#1 and #2 are positive about it, ref#3 is more critical. As such, we would like you to experimentally show the direct role of VPA through NS1 acetylation as suggested by ref#1 and #3. In addition, ref#2 is concerned by the high titers of viruses and would be convinced of enhanced replication, spread and oncolysis if increased viral titres could be shown in isolated tumours. Finally, ref#3 has several important criticisms, however we would like you to particularly focus on testing another HDAC inhibitor in a key experiment and different doses of VPA. If you have data on hand addressing the other concerns of ref#3, we would strongly encourage you to include these in the manuscript.

Should you be able to address the raised concerns with additional experiments where appropriate, we would be willing to consider a revised manuscript.

Please note that it is EMBO Molecular Medicine policy to allow a single round of revision in order to avoid the delayed publication of research findings. Consequently, acceptance or rejection of the

manuscript will depend on the completeness of your responses included in the next version of the manuscript.

EMBO Molecular Medicine has a "scooping protection" policy, whereby similar findings that are published by others during review or revision are not a criterion for rejection. Should you decide to submit a revised version, I would like to ask you to get in touch after three months if you have not completed it, to update us on the status.

Please also contact us as soon as possible if similar work is published elsewhere. If other work is published we may not be able to extend the revision period beyond three months.

I look forward to receiving your revised manuscript.

***** Reviewer's comments *****

Referee #1:

This paper reports a remarkably comprehensive study of the use of the histone deacetylase inhibitor (HDAC) valproic acid (VPA) in combination therapy with the oncolytic parvovirus H-1PV, against cervical and pancreatic cells both in culture and as xenografts in immnuodeficient rats and mice.

The analysis in cultured cells is extensive, well-documented and reveals, rather unexpectedly, that the major non-structural protein of H-1PV is acetylated on lysine residues, and that the level of acetylation is increased by the HDAC inhibitor, implying that the NS1 of H-1PV may be recognized by the epigenetic maintenance mechanisms operating in its host cell.

The authors go on to map the two lysine residues that are acetylated, and use reverse genetics and antibody pull-down experiments to confirm that these are the major sites of acetylation, and that, in turn, their de-acetylation is inhibited by VPA. This is an exciting, and unpredicted, new insight into the biology of the parvoviruses that will surely lead to a greater understanding of the role of the multifunctional NS1 protein in viral replication, oxidative stress induction and resulting oncolytic activity. In this respect, the authors show, very convincingly, that inhibiting the de-acetylation of these two residues with VPA leads to a synergistic elevation of viral DNA replication, progeny formation and oncolytic activity of H-1PV, both in vitro and in vivo.

Indeed, the authors show that combined therapy with intermediate doses of H-1PV and low concentrations of VPA can achieve complete remission in a number of human xenograft tumour models. That the majority of these studies were done in nude rats, the rat being the natural host species of H-1PV, goes some way to alleviating many of the concerns associated with using immunodeficient rodent xenograft models with oncolytic viruses that do not infect the host species. The finding that "cured" immnunodeficient rats not only survive the H1-PV infection, but remain healthy and tumour-free for more than a year following treatment, is very encouraging.

In general, the manuscript is very clearly and tightly written and the authors' conclusions are well-supported by the data presented. There are a small number of minor points that the authors should consider that would benefit from clarification:-

Page 2, line 11: to what extent are the K85 and K257 residues conserved across the parvoviruses?

Page 3, line 18: Hristov et al., 2010?

Page 7, line 2: "hall marker" = "hallmark"?

Page 8, line 15: although quantitation is not provided for NS1, it appears that acetylation does not affect the level of NS1 produced during infection - although it is known to upregulate its own, P4,

promoter as well as p38. This might be discussed.

Page 9, line 5: substituting R for K at these two positions does not "mimic the non-acetylated state" - it conserves the basic nature of the residue while presumably eliminating its ability to be acetylated.

Page 12, line 17: "culminating" = "combining"?

Page 13, line 11-12: the direct role for VPA through NS1 acetylation could be tested using K85R;K257R double mutant H-1PV virus, for which oncolysis should not be enhanced by VPA if this is the sole effect of the HDAC inhibitor.

Page 18, line 13: what is "HMGS"?

Page 38, line 6-7: this citation is incomplete.

Page 42, line 16: "acetylated in" = "acetylated at"?

Page 42, line 19: "trypsin-digested"

Fig 7: panel A - the key should be outside the plot - its current location makes this tiny figure even more difficult to see!

Page S1, legend to Fig S2: since RT-qPCR is the output for these infections, why use a recombinant H-1PV, and not just monitor the P4 transcripts of the wildtype virus?

Page S2, legend to Figs S4 & S5: what animals - and what tumours? Presumably HeLa in Fig S5, but what in Fig S4?

Page S3, legend to Fig S7: aren't the tumours in Fig S5 HeLa-derived, not AsPC-1 - or do these PDAC cells also contain HPV18 sequences?

Page S3, legend to Fig S8: what "analysis" was performed "after 28 days"?

Referee #2:

Several aspects of this study call into question the claim that the HACi increases viral replication (by modifying directly NS1 and enhancing viral gene expression and replication).

In Fig1A rather high MOI are used in several cases implying that the virus is not able to spread in these cultures but rather is toxic if enough particles contact cells.

In Fig4 a ten-fold change in MOI does not appreciably increase the titre produced, are these viral titres measured at plateau and so MOI does not matter? and then does VPA actually increase the plateau/maximal titre achieved?

When using a rather resistant cell line (AsPC-1) in vivo the authors still achieve tumour regressions, but the evidence provided for enhanced viral infection of tumours is not convincing (Fig 9c). If it is true that there is enhanced replication, spread and oncolysis this should be demonstrated by increased viral titres in isolated tumours. Otherwise it is tempting to assume that the effects are largely due to some sort of enhanced bystander effect (possible due to innate immune mechanisms).

Referee #3:

The manuscript by Li, J. et al. describes a rather thorough study demonstrating synergy between valproic acid and the rat H-1PV oncolytic virus in cervical and pancreatic carcinoma cell models. The studies are technically and methodologically robust and logical conclusions are presented.

Therefore, the quality of the manuscript is very good (besides some language issues) and would be suitable for publication. However, the findings are hardly novel and the proposed mechanism for the synergistic effects is not conclusively verified.
HDAC inhibitors have been explored in combination with numerous oncolytic mutants and been demonstrated to almost universally increase virus potency through both virus and cell line dependent properties. Furthermore, only one HDAC inhibitor (VPA) is tested and only 2 out of 14 Lys-residues in the NS1 protein are investigated and suggested as responsible for both viral gene expression, replication and synergistic cell killing. Despite generation of stably expressing HeLa cells with the two lysine residues mutated to eliminate acetylation, it is not clear that this is the mechanism for the synergistic effects. How about acetylation of NS1? What cellular effects does VPA have - it is a very high dose (1mM)? How about other HDAC inhibitors?
Based on these concerns I cannot recommend publication of the manuscript including the current data in Embo Molecular Therapy.

Specific comments:

The statement in the introduction "......tumour relapse due to the emergence of virus-resistant cancer cells." is mis-leading. To my knowledge, 'classical' resistance does not apply to most oncolytic viruses. Some cells are not sensitive to virus but emergence of resistant cells in the course of virus-treatment has not been reported. Does this phrase refer specifically to H-1PV?

The use of a dose of 1mM VPA seems extremely high and hardly within pharmacological applications. Please explain.

In figure 1, why is not viral replication measured in each cell line? Lysis could be caused by expression of cytotoxic genes alone. Only in fig. 4 is replication measured and only in HeLa cells.

In figure 3, the increase in NS1 acetylation by VPA is extremely faint and so is the VP induction (not significant?). It is hard to believe that this is the cause of the increased cytotoxicity. Why were not acetylation induced?

1st Revision - authors' response 23 July 2013

Thank you for sending us your comments and those of the Reviewers about our manuscript entitled “Synergistic combination of Valproic acid and oncolytic parvovirus H-1PV as a potential therapy against cervical and pancreatic carcinomas”. We were pleased to receive positive feedback and grateful for the suggestions given to improve our manuscript. We have now successfully performed all the additional experiments requested. All new results substantiate our previous data indicating that HDACIs enhance H-1PV oncolysis by multiple mechanisms including: 1) the induction of oxidative stress leading to increased DNA damage, apoptosis and cell lysis; 2) the increase in NS1 acetylation leading to a larger virus production in permissive tumor cells. For the sake of clarity, we found it useful to include an additional figure **(new Fig. 10)** summarizing the results described in the present study in the form of a tentative model.

We thank you and the reviewers for the constructive criticisms and we hope that our manuscript now fulfills all the requirements for publication in EMBO Molecular Medicine.

Please find below our point-to point rebuttal letter to your and reviewers’ concerns:

Your comments:

Thank you for the submission of your manuscript to EMBO Molecular Medicine. We have now heard back from the three referees whom we asked to evaluate your manuscript. Although the referees find the study to be of potential interest, they also raise a number of concerns that need to be addressed in a major revision of your manuscript.

As you will see from the enclosed reports, the referees find the study interesting and comprehensive.

While ref#1 and #2 are positive about it, ref#3 is more critical. As such, we would like you to experimentally show

1.) the direct role of VPA through NS1 acetylation as suggested by ref#1 and #3.

AUTHORS: As mentioned above, the present study revealed two mechanisms through which Valproic acid (VPA) enhances PV oncolytic activities. However, there may still be other mechanisms by which VPA could stimulate virus cytotoxicity. In particular, it has been reported that HDACI treatment affects the expression of about 10% of all cellular genes. It is therefore conceivable that some of the genes affected by VPA encode factors controlling virus replication and/or cytotoxicity. On the basis of our and others data, the mechanisms by which VPA may boost H-1PV replication and cytotoxicity have been extensively discussed in the new version of the manuscript on pages 13 and 14 (revised discussion section).

2) In addition, ref#2 is concerned by the high titters of viruses and would be convinced of enhanced replication, spread and oncolysis if increased viral titres could be shown in isolated tumours.

*AUTHORS: We agreed with this comment and performed new experiments using both HeLa and AsPC-1 xenograft rat models, in which viral production was measured in isolated tumours. In both animal models, we confirmed that VPA treatment increases viral protein levels and infectious virus production (**new Fig 8 D and E and new Fig. 9 D and E**).*

3) Finally, ref#3 has several important criticisms, however we would like you to particularly focus on testing another HDAC inhibitor in a key experiment and different doses of VPA.

AUTHORS: The combination of H-1PV with a second HDAC inhibitor, namely sodium butyrate (NaB) has also been tested and is reported in the revised version of the manuscript. A series of new experiments was performed in HeLa cells showing that the NaB treatment acts like VPA in that it increases:

*i) H-1PV mediated cell lysis via generation of ROS and DNA damage (**new Supp. Fig 3**)*
*ii) NS1 intrinsic cytotoxicity (**new Supporting Information Fig S4**)*
*iii) NS1 acetylation (**new Fig. 3A and new Fig 5A**)*
*iv) NS1 transcriptional activity (**new Supporting Information Fig S5**)*

***VPA has been also tested at the concentration of 0.5 mM** in all seven cell lines analysed in this study, confirming previous results obtained using 1mM VPA (**new Supportive Information Fig S1 and revised Table S1**). We also explained that both 0.5 and 1mM doses were selected based on the concentrations that have been established in patients with epilepsy: 0.5 mM (close to the typical therapeutic serum concentration of 0.6mM) and 1mM (close to the upper limit of antiepilectic range of 0.9 mM). This information is provided on page 6 lines 106-109 together with references.*

4) If you have data on hand addressing the other concerns of ref#3, we would strongly encourage you to include these in the manuscript.

AUTHORS: Please refer to our response to reviewer 3 about our efforts to address his/her additional concerns.

***** Reviewer's comments *****

Referee #1:

This paper reports a remarkably comprehensive study of the use of the histone deacetylase inhibitor (HDAC) valproic acid (VPA) in combination therapy with the oncolytic parvovirus H-1PV, against cervical and pancreatic cells both in culture and as xenografts in immnuodeficient rats and mice.

The analysis in cultured cells is extensive, well-documented and reveals, rather unexpectedly, that the major non-structural protein of H-1PV is acetylated on lysine residues, and that the level of acetylation is increased by the HDAC inhibitor, implying that the NS1 of H-1PV may be recognized by the epigenetic maintenance mechanisms operating in its host cell.

The authors go on to map the two lysine residues that are acetylated, and use reverse genetics and antibody pull-down experiments to confirm that these are the major sites of acetylation, and that, in turn, their de-acetylation is inhibited by VPA. This is an exciting, and unpredicted, new insight into the biology of the parvoviruses that will surely lead to a greater understanding of the role of the multifunctional NS1 protein in viral replication, oxidative stress induction and resulting oncolytic activity. In this respect, the authors show, very convincingly, that inhibiting the de-acetylation of these two residues with VPA leads to a synergistic elevation of viral DNA replication, progeny formation and oncolytic activity of H-1PV, both in vitro and in vivo.

Indeed, the authors show that combined therapy with intermediate doses of H-1PV and low concentrations of VPA can achieve complete remission in a number of human xenograft tumour models. That the majority of these studies were done in nude rats, the rat being the natural host species of H-1PV, goes some way to alleviating many of the concerns associated with using immunodeficient rodent xenograft models with oncolytic viruses that do not infect the host species. The finding that "cured" immnunodeficient rats not only survive the H1-PV infection, but remain healthy and tumour-free for more than a year following treatment, is very encouraging.

In general, the manuscript is very clearly and tightly written and the authors' conclusions are well-supported by the data presented.

AUTHORS: We thank the reviewer for the kind comments. We were happy to know that he/she found our paper interesting and well written.

There are a small number of minor points that the authors should consider that would benefit from clarification:

Page 2, line 11: to what extent are the K85 and K257 residues conserved across the parvoviruses?

AUTHORS: This information has been included in the discussion on page 15 lines 348 353.

Page 3, line 18: Hristov et al., 2010?

AUTHORS: Corrected. Thank you

Page 7, line 2: "hall marker" = "hallmark"?

AUTHORS: Corrected. Thank you

Page 8, line 15: although quantitation is not provided for NS1, it appears that acetylation does not affect the level of NS1 produced during infection - although it is known to upregulate its own, P4, promoter as well as p38. This might be discussed.

AUTHORS: In this study the ability of NS1 to activate its own P4 promoter in the presence or absence of VPA has been not investigated in details. While cell culture experiments using HeLa cells suggest that VPA treatment does not result in a significant change in NS1 protein levels during H-1PV infection, experiments in animals show a clear induction. We feel that further studies are required to clarify this issue. This point is mentioned in the discussion on page 15 lines 346 and 347.

Page 9, line 5: substituting R for K at these two positions does not "mimic the non-acetylated state" - it conserves the basic nature of the residue while presumably eliminating its ability to be acetylated.

AUTHORS: Thank you for this comment. We have revised the text accordingly (line 202).

Page 12, line 17: "culminating" = "combining"?

AUTHORS: we have revised the entire sentence to make it clearer (Page 13 lines 304-305).

Page 13, line 11-12: the direct role for VPA through NS1 acetylation could be tested using

K85R;K257R double mutant H-1PV virus, for which oncolysis should not be enhanced by VPA if this is the sole effect of the HDAC inhibitor.

AUTHORS: we have addressed this question above (our response to the editor's comments, point 1).

Page 18, line 13: what is "HMGS"?

AUTHORS: the abbreviation has been explained (line 456).

Page 38, line 6-7: this citation is incomplete.

AUTHORS: Corrected. Thank you.

Page 42, line 16: "acetylated in" = "acetylated at"?

AUTHORS: Corrected. Thank you.

Page 42, line 19: "trypsin-digested"

AUTHORS: Corrected. Thank you.

Fig 7: panel A - the key should be outside the plot - its current location makes this tiny figure even more difficult to see!

AUTHORS: We have revised the figure accordingly.

Page S1, legend to Fig S2: since RT-qPCR is the output for these infections, why use a recombinant H-1PV, and not just monitor the P4 transcripts of the wildtype virus?

AUTHORS: The purpose of this experiment was not to monitor the activity of the parvovirus P4 promoter. The recPV-GFP virus contains the GFP gene placed under the control of the parvovirus P38 promoter which is trans-activated by NS1. This vector was thus used to confirm that in the presence of VPA there is an increase in NS1 transcriptional activity. Results are in agreement with data obtained by dual luciferase assay and with the VPA-dependent increase in VP1 and VP2 protein levels observed upon H-1PV infection.

Page S2, legend to Figs S4 & S5: what animals - and what tumours? Presumably HeLa in Fig S5, but what in Fig S4?
Page S3, legend to Fig S7: aren't the tumours in Fig S5 HeLa-derived, not AsPC-1 - or do these PDAC cells also contain HPV18 sequences?
Page S3, legend to Fig S8: what "analysis" was performed "after 28 days"?

AUTHORS: All the three figure legends have been revised according to the referee's suggestions. Thank you.

Referee #2:

Several aspects of this study call into question the claim that the HACi increases viral replication (by modifying directly NS1 and enhancing viral gene expression and replication).

In Fig1A rather high MOI are used in several cases implying that the virus is not able to spread in these cultures but rather is toxic if enough particles contact cells.

AUTHORS: We agree with the reviewer. Among the cell lines used only HeLa cells sustain efficient viral multiplication, while the other cell lines tested are low permissive for virus replication (our unpublished results and Dempe et al. IJC 2010). This information is provided in the text on page 6 lines 104-106.

In Fig4 a ten-fold change in MOI does not appreciably increase the titre produced, are these viral titres measured at plateau and so MOI does not matter? and then does VPA actually increase the plateau/maximal titre achieved?

*AUTHORS: We thank the reviewer for this comment which gave us the possibility to examine in greater detail H-1PV production in HeLa cells. We carried out a time course experiment in which virus production and release was monitored every day for a total of five days. Results from this experiment confirmed that the presence of VPA stimulates both virus production and release. Interestingly, while viral titters in the absence of VPA reached a plateau after 72 hours, VPA stimulated virus production beyond that limit. This observation has been included in the new version of the manuscript on page 9 lines 179-182 and in the **new Fig 4B**.*

When using a rather resistant cell line (AsPC-1) in vivo the authors still achieve tumour regressions, but the evidence provided for enhanced viral infection of tumours is not convincing (Fig 9c). If it is true that there is enhanced replication, spread and oncolysis this should be demonstrated by increased viral titres in isolated tumours. Otherwise it is tempting to assume that the effects are largely due to some sort of enhanced bystander effect (possible due to innate immune mechanisms).

AUTHORS: Please see our answer to the Editor's comments, point 2.

Referee #3:

The manuscript by Li, J. et al. describes a rather thorough study demonstrating synergy between valproic acid and the rat H-1PV oncolytic virus in cervical and pancreatic carcinoma cell models. The studies are technically and methodologically robust and logical conclusions are presented. Therefore, the quality of the manuscript is very good (besides some language issues) and would be suitable for publication. However, the findings are hardly novel and the proposed mechanism for the synergistic effects is not conclusively verified.
HDAC inhibitors have been explored in combination with numerous oncolytic mutants and been demonstrated to almost universally increase virus potency through both virus and cell line dependent properties.

AUTHORS: We only partly agree with the reviewer's comment. While oncolytic viruses have been indeed tested in combination with various HDAC inhibitors as duly reported in our introduction and discussion, we still believe that the synergistic oncosuppressive effect of the H-1PV/HDACI combination against cervical and pancreatic carcinomas was not predictable from the literature and represents an original discovery. Besides improving substantially parvovirus cytotoxicity, this combination allowed for the first time full tumour remission to be achieved. To the best of our knowledge, no similar achievement has been reported for other oncolytic viruses in the animal models used in the present study. Furthermore, we unravelled a completely novel mechanism of regulation of the cytotoxic NS1 protein by discovering that NS1 is acetylated and that VPA increases the acetylation status of the protein and thereby enhances parvovirus cytotoxicity. This finding not only improves our knowledge of parvovirus biology, but has also important implications regarding the clinical use of this virus in cancer therapy.

Furthermore, only one HDAC inhibitor (VPA) is tested and only 2 out of 14 Lys-residues in the NS1 protein are investigated and suggested as responsible for viral gene expression, replication and synergistic cell killing.
*AUTHORS: There is a misunderstanding on the number of Lys-residues investigated, which is by far higher than 2. Our analysis actually covered 74.2% of the entire NS1 sequence including 36 out of 48 Lys residues (**see Fig. 5B**) Only 12 lysines were not tested by our MS analysis. This has been now explained in greater detail on page 9 line 199 (results section) and on page 15 lines 360-361 (discussion section).*

Despite generation of stably expressing HeLa cells with the two lysine residues mutated to eliminate acetylation, it is not clear that this is the mechanism for the synergistic effects. How about acetylation of NS1?
What cellular effects does VPA have - it is a very high dose (1mM)?

AUTHORS: As mentioned above, the VPA concentration of 1mM used in our cell culture experiments is close to the upper limit of the therapeutic concentration range (0.6-0.9 mM) established in patients with epilepsy (see our answer to the editor's comments, point 3). Nevertheless, to ascertain the relevance of our results, we have followed the Editor's suggestion and repeated LDH experiments using the VPA at a 0.5 mM concentration. Under these conditions also improved H-1PV oncolysis could be demonstrated in the presence of VPA. It is also noteworthy that the VPA concentration used in animal experimentation (100 mg/kg) corresponds to the human equivalent of 16 mg/kg as calculated according to the body surface area normalization method recommended by the Food and Drug Administration for conversion of drug doses between species (Reagan-Shaw et al, 2008). This dose is within the clinical range of 15-30 mg/kg used for long-term treatment of epileptic patients and far below the limit of 60 mg/kg considered safe and well tolerated in humans (Atmaca et al, 2007). This important information has been added in our manuscript on page 19-20 lines 461 -467 (materials and methods section).

How about other HDAC inhibitors?

AUTHORS: As suggested by the Editor and this Reviewer a full series of experiments has been repeated using a second HDAC inhibitor, namely NaB. These data have now been included in the revised version of the manuscript (please see our answer to the Editor's comments, point 3).

Based on these concerns I cannot recommend publication of the manuscript including the current data in Embo Molecular Therapy.

AUTHORS: We hope that the additional data included in this revised version will convince this reviewer that our manuscript is suitable for publication in EMM. In our opinion, we have addressed all his/her concerns.

Specific comments:
The statement in the introduction "......tumour relapse due to the emergence of virus-resistant cancer cells." is mis-leading. To my knowledge, 'classical' resistance does not apply to most oncolytic viruses. Some cells are not sensitive to virus but emergence of resistant cells in the course of virus-treatment has not been reported. Does this phrase refer specifically to H-1PV?

AUTHORS: Emergence of resistant cells in the course of parvovirus treatment has been reported under in vitro conditions. Its occurrence in vivo has not indeed been described so far. However, this possibility deserves in our opinion, to be considered due to the high mutation rate of cancer cells. As the parvovirus relies on host cell factors for its replication and cytotoxicity, inactivating mutations in some of the genes encoding these factors may result in acquisition of resistance.

The use of a dose of 1mM VPA seems extremely high and hardly within pharmacological applications. Please explain.

AUTHORS: See our answer above.

In figure 1, why is not viral replication measured in each cell line? Lysis could be caused by expression of cytotoxic genes alone. Only in fig. 4 is replication measured and only in HeLa cells.

*AUTHORS: It was beyond the initial scope of this study to investigate viral replication in all the cancer cell lines used. We still took this comment into consideration and extended our analysis to two other cervical carcinoma cell lines (SiHa and CaSki). As in the case of HeLa cells, an increase in parvovirus replication was also observed in these cells upon VPA treatment (**New Supportive Information Fig S6**). Moreover, enhanced virus replication has been also demonstrated in vivo in tumours from VPA-treated animals (see our response to the Editor's comments, point 2).*

In figure 3, the increase in NS1 acetylation by VPA is extremely faint and so is the VP induction (not significant?). It is hard to believe that this is the cause of the increased cytotoxicity. Why were not acetylation induced?

AUTHORS: As documented, in the new version of the manuscript we tested the levels of NS1

*acetylation in the presence and absence of VPA at two different time points (16 and 32 hours) and showed that VPA treatment enhances NS1 acetylation in a time-dependent manner (**New figure 3A**).*

2nd Editorial Decision 05 August 2013

Thank you for the submission of your revised manuscript to EMBO Molecular Medicine. We have now heard back from the two Reviewers whom we asked to evaluate your manuscript.

You will see that while Reviewer 1 is supportive of you work, Reviewer 3 has one remaining issue that requires your action before we can accept your manuscript for publication.

Please fully address the Reviewer 3's concern as quickly as possible and in any case within two weeks. Provided these issues are fully addressed, the final decision will be made at the Editorial level.

I look forward to receiving your re-revised manuscript as soon as possible and in any case within two weeks.

***** Reviewer's comments *****

Referee #1 (Comments on Novelty/Model System):

The authors have satisfactorily addressed all of this reviewer's concerns.

Referee #1 (General Remarks):

In revising their manuscript, the authors have significantly improved an already comprehensive and ground-breaking study

Referee #3 (General Remarks):

The current version of the manuscript is much improved and in my opinion it interesting and should be published. However, I leave it for the editor to decide whether it is suitable for publication in EMBO Molecular Medicine.
The data appear to be of high quality, reproducible and several key findings were verified in more than one model system and with two different HDAC inhibitors. While evidence supporting the claims in the title and abstract are clear, evidence of a direct mechanism (through Lys-acetylation of the virus) for the increased cell killing when virus and drugs are combined are not completely convincing only strongly indicated.
Most of this reviewer's queries and comments from the previous version have been addressed in the revised manuscript except the following that need clarification to avoid misunderstanding:

Line 68-71: This makes necessary to reinforce the antineoplastic activities of H-1PV to make it more effective. For this, two approaches might be used: sensitizing the tumour cells to parvovirus cytotoxicity and/or killing virus-resistant tumour cells by other means.

Comment: The authors' statement is still misleading since the impression is that they are talking about tumor-tissue properties in the clinical setting not in cultured cell lines. In the response to my comments in the previous review, they agreed that viral resistance (using the same definition as for drug-resistance) has not been demonstrated in vivo only in cultured cell lines. Therefore, this should be specified in the introduction. Resistance is a difficult term to define and possible misunderstanding should be avoided.

2nd Revision - authors' response 05 August 2013

Referee #1 (Comments on Novelty/Model System):

The authors have satisfactorily addressed all of this reviewer's concerns.

Referee #1 (General Remarks):

In revising their manuscript, the authors have significantly improved an already comprehensive and ground-breaking study

AUTHORS: Thank you very much for your kind comments.

Referee #3 (General Remarks):

The current version of the manuscript is much improved and in my opinion it interesting and should be published. However, I leave it for the editor to decide whether it is suitable for publication in EMBO Molecular Medicine.
The data appear to be of high quality, reproducible and several key findings were verified in more than one model system and with two different HDAC inhibitors. While evidence supporting the claims in the title and abstract are clear, evidence of a direct mechanism (through Lys-acetylation of the virus) for the increased cell killing when virus and drugs are combined are not completely convincing only strongly indicated.

AUTHORS: As mentioned above we were happy to know that the reviewer found our manuscript improved and interesting.

Most of this reviewer's queries and comments from the previous version have been addressed in the revised manuscript except the following that need clarification to avoid misunderstanding:

Line 68-71: This makes necessary to reinforce the antineoplastic activities of H-1PV to make it more effective. For this, two approaches might be used: sensitizing the tumour cells to parvovirus cytotoxicity and/or killing virus-resistant tumour cells by other means.
Comment: The authors' statement is still misleading since the impression is that they are talking about tumour-tissue properties in the clinical setting not in cultured cell lines. In the response to my comments in the previous review, they agreed that viral resistance (using the same definition as for drug-resistance) has not been demonstrated in vivo only in cultured cell lines. Therefore, this should be specified in the introduction. Resistance is a difficult term to define and possible misunderstanding should be avoided.

AUTHORS: To avoid any possible misunderstanding with the word resistance we have decided to substitute the old sentence (...In the framework of cancer therapy and as also observed with other oncolytic viruses, there is still a risk of tumour relapse due to the presence of parvovirus-resistant cancer cells. This makes necessary to reinforce the antineoplastic activities of H-1PV to make it more effective. For this, two approaches might be used: sensitizing the tumor cells to parvovirus cytotoxicity and/or killing virus-resistant tumor cells by other means) with this new one:

"Due to their genetic heterogeneity, it is likely that some of the cancer cells within a tumour will have a different sensitivity to H-1PV. It is therefore important to reinforce the antineoplastic activity of the virus in order to improve its clinical outcome in such a scenario.
This can be achieved by developing combination strategies based on virus and other anticancer agents that increase cancer cell killing while minimizing toxic side effects." (introduction, lines 67-71).

© EMBO
